# Access to antiretroviral therapy in HIV-infected children aged 0–19 years in the International Epidemiology Databases to Evaluate AIDS (IeDEA) Global Cohort Consortium, 2004–2015: A prospective cohort study

**DOI:** 10.1371/journal.pmed.1002565

**Published:** 2018-05-04

**Authors:** Sophie Desmonde, Franck Tanser, Rachel Vreeman, Elom Takassi, Andrew Edmonds, Pagakrong Lumbiganon, Jorge Pinto, Karen Malateste, Catherine McGowan, Azar Kariminia, Marcel Yotebieng, Fatoumata Dicko, Constantin Yiannoutsos, Mwangelwa Mubiana-Mbewe, Kara Wools-Kaloustian, Mary-Ann Davies, Valériane Leroy, for the International Epidemiology Databases to Evaluate AIDS (IeDEA) Pediatric Working Group

**Affiliations:** 1 Inserm U1027, Toulouse III University, Toulouse, France; 2 Africa Centre for Health and Population Studies, University of KwaZulu-Natal, Somkhele, South Africa; 3 School of Medicine, Indiana University, Indianapolis, Indiana, United States of America; 4 CHU Sylvanus Olympio, Lomé, Togo; 5 Department of Epidemiology, University of North Carolina at Chapel Hill, Chapel Hill, North Carolina, United States of America; 6 Khon Kaen University, Khon Kaen, Thailand; 7 School of Medicine, Universide Federal de Minas Gerais, Belo Horizonte, Brazil; 8 Inserm U1219, University of Bordeaux, Bordeaux, France; 9 Bordeaux School of Public Health, University of Bordeaux, Bordeaux, France; 10 Division of Infectious Diseases, Vanderbilt University Medical Center, Nashville, Tennessee, United States of America; 11 Kirby Institute, University of New South Wales, Sydney, New South Wales, Australia; 12 Division of Epidemiology, College of Public Health, Ohio State University, Columbus, Ohio, United States of America; 13 Hopital Gabriel Touré, Bamako, Mali; 14 Richard M. Fairbanks School of Public Health, Indiana University, Indianapolis, Indiana, United States of America; 15 Centre for Infectious Disease Research in Zambia, Lusaka, Zambia; 16 Centre for Infectious Disease Epidemiology and Research, School of Public Health and Family Medicine, University of Cape Town, Cape Town, South Africa; Elizabeth Glaser Pediatric AIDS Foundation, UNITED STATES

## Abstract

**Introduction:**

Access to antiretroviral therapy (ART) is a global priority. However, the attrition across the continuum of care for HIV-infected children between their HIV diagnosis and ART initiation is not well known. We analyzed the time from enrollment into HIV care to ART initiation in HIV-infected children within the International Epidemiology Databases to Evaluate AIDS (IeDEA) Global Cohort Consortium.

**Methods and findings:**

We included 135,479 HIV-1-infected children, aged 0–19 years and ART-naïve at enrollment, between 1 January 2004 and 31 December 2015, in IeDEA cohorts from Central Africa (3 countries; *n =* 4,948), East Africa (3 countries; *n =* 22,827), West Africa (7 countries; *n =* 7,372), Southern Africa (6 countries; *n =* 93,799), Asia-Pacific (6 countries; *n =* 4,045), and Latin America (7 countries; *n =* 2,488). Follow-up in these cohorts is typically every 3–6 months. We described time to ART initiation and missed opportunities (death or loss to follow-up [LTFU]: last clinical visit >6 months) since baseline (the date of HIV diagnosis or, if unavailable, date of enrollment). Cumulative incidence functions (CIFs) for and determinants of ART initiation were computed, with death and LTFU as competing risks. Among the 135,479 children included, 99,404 (73.4%) initiated ART, 1.9% died, 1.4% were transferred out, and 20.4% were lost to follow-up before ART initiation. The 24-month CIF for ART initiation was 68.2% (95% CI: 67.9%–68.4%); it was lower in sub-Saharan Africa—ranging from 49.8% (95% CI: 48.4%–51.2%) in Central Africa to 72.5% (95% CI: 71.5%–73.5%) in West Africa—compared to Latin America (71.0%, 95% CI: 69.1%–72.7%) and the Asia-Pacific (78.3%, 95% CI: 76.9%–79.6%). Adolescents aged 15–19 years and infants <1 year had the lowest cumulative incidence of ART initiation compared to other ages: 62.2% (95% CI: 61.6%–62.8%) and 66.4% (95% CI: 65.7%–67.0%), respectively. Overall, 49.1% were ART-eligible per local guidelines at baseline, of whom 80.6% initiated ART. The following children had lower cumulative incidence of ART initiation: female children (*p <* 0.01); those aged <1 year, 2–4 years, 5–9 years, and 15–19 years (versus those aged 10–14 years, *p <* 0.01); those who became eligible during follow-up (versus eligible at enrollment, *p <* 0.01); and those receiving care in low-income or lower-middle-income countries (*p <* 0.01). The main limitations of our study include left truncation and survivor bias, caused by deaths of children prior to enrollment, and use of enrollment date as a proxy for missing data on date of HIV diagnosis, which could have led to underestimation of the time between HIV diagnosis and ART initiation.

**Conclusions:**

In this study, 68% of HIV-infected children initiated ART by 24 months. However, there was a substantial risk of LTFU before ART initiation, which may also represent undocumented mortality. In 2015, many obstacles to ART initiation remained, with substantial inequities. More effective and targeted interventions to improve access are needed to reach the target of treating 90% of HIV-infected children with ART.

## Abstract 2

In a research article, Valériane Leroy and colleagues study access to antiretroviral therapy in a large population of children and adolescents with HIV-1 infection across 6 regions, and identify priorities for improving access to treatment.

## Author summary

**Why was this study done?:**

- Access to antiretroviral therapy (ART) has been highlighted as an urgent global priority area by a diverse range of stakeholders, including the World Health Organization, the Joint United Nations Programme on HIV/AIDS (UNAIDS), and, more specifically for children, the United Nations Children’s Fund.
- In 2014, UNAIDS set the ambitious 90-90-90 targets that, by 2020, 90% of people living with HIV should know their HIV status, 90% of HIV-infected people who know their HIV status should receive antiretroviral treatment, and 90% of people on treatment should be virologically suppressed.
- In low/middle-income countries, the attrition across the continuum of care for HIV-infected children between their HIV diagnosis, linkage to care, and ART initiation is not well characterized.

**What did the researchers do and find?:**

- We used the International Epidemiological Databases to Evaluate AIDS (IeDEA), bringing together data from 6 different regions worldwide (in Asia-Pacific, sub-Saharan Africa, and Latin America), including the regions most affected by the HIV epidemic, to describe access to pediatric ART since the scaling up of ART.
- We described the cumulative incidence of and time to ART initiation in HIV-infected children in care between 2004 and 2015 (*n* = 135,479) according to region, sex, age, and eligibility criteria at baseline, since enrollment in care or HIV diagnosis, when this was available.
- The global probability for initiating ART within 2 years in care was 68%; 20% of the children were lost to follow-up, and 2% died before initiating treatment.
- ART initiation rates were lower in sub-Saharan Africa compared to the other regions, and children aged <1 year and those aged 15–19 years were the least likely to initiate treatment.
- Overall, 49% of the children were eligible for ART at baseline, and 81% of these children initiated treatment.

**What do these findings mean?:**

- In many low- and middle-income countries, obstacles to ART initiation for children remained, with substantial inequities, in 2015.
- More effective and targeted interventions to improve access are needed to reach the target of treating 90% of children with ART.
- With adoption of universal treatment recommendations since 2015, it will be crucial to further monitor progress and identify gaps in ART coverage to achieve the 90-90-90 targets for children and adolescents.

## Introduction

By the end of 2016, the Joint United Nations Programme on HIV/AIDS (UNAIDS) estimated that 2.1 million children aged <15 years were living with HIV worldwide [1]. Despite effective interventions for the prevention of mother-to-child transmission, the pediatric epidemic persists, and an estimated 160,000 children were newly infected with HIV in 2016 [1]. Furthermore, the incidence of HIV remains alarmingly high in adolescents and young people aged 15–24 years. According to UNAIDS, 37% of new HIV infections occurring in sub-Saharan African adults in 2016 were among this population [1].

In the absence of timely antiretroviral therapy (ART), mortality in HIV-infected children reaches 52% by the age of 2 years [2]. Systematic early ART initiation in infants <3 months of age has proven an effective intervention in reducing early infant mortality, and the World Health Organization (WHO) has recommended ART in all HIV-infected children aged <2 years since 2010; in 2013, the recommendation to initiate ART regardless of clinical stage or CD4 count was extended to children <5 years [3–5]. In 2015, guidelines were revised to recommend ART initiation regardless of age, clinical, or immunological criteria [6]. However, in 2015, 49% of children aged <15 years who were eligible for ART based on previous guidelines were still not receiving treatment worldwide [7]. Many of those children who did not initiate ART either had no access to treatment or had unknown HIV status, mainly through lack of access to early HIV diagnosis.

In 2014, UNAIDS set the ambitious targets that, by 2020, 90% of people living with HIV should know their HIV status, 90% of people who know their HIV status should receive treatment, and 90% of people on treatment should be virologically suppressed [8]. However, in low-income and middle-income countries, attrition across the continuum of care for HIV-infected children between their HIV diagnosis and ART initiation is not well known. The period between diagnosis of HIV infection and ART initiation, which includes periods from testing to linkage to care and then from inclusion in care to ART initiation, is known as the pre-ART cascade. Throughout the pre-ART period, children are at risk for attrition due to death and loss to follow-up (LTFU). A better understanding of the attrition of children across the pre-ART cascade is crucial to reach the 90-90-90 targets, particularly for the second target, to initiate ART in 90% of HIV-infected children identified, assuming that 90% have been identified among those HIV infected.

In 2006, the US National Institutes of Health launched the International Epidemiology Databases to Evaluate AIDS (IeDEA) to describe trends in HIV epidemiology in the context of ART access across regions of the world (https://www.iedea.org/). Clinics from 7 international regional data centers contribute both retrospective cohort data, prior to 2006, and prospective data since, on care and treatment of HIV to evaluate the outcomes of people living with HIV/AIDS. To better understand the continuum of care from HIV diagnosis to ART initiation in HIV-infected children and in collaboration with WHO, we performed a multiregional analysis of the pre-ART retention cascade of HIV-infected children from HIV diagnosis to ART initiation within IeDEA from 2004 to 2015.

## Methods

### Study design and population

This multiregional analysis was prespecified, except for the sensitivity analysis, in an approved concept plan available in the Supporting Information (S1 Concept Plan). We pooled individual patient data from 6 pediatric cohorts of IeDEA and included clinical care sites from Asia-Pacific, West Africa, East Africa, Central Africa, Southern Africa, and Latin America. We included all HIV-infected children aged 0–19 years at enrollment into any IeDEA-affiliated pediatric care program who were ART-naïve at enrollment (except for exposure to perinatal prevention of mother-to-child transmission prophylaxis) between 1 January 2004 (corresponding to the beginning of the era of ART access in low-income countries) and 31 December 2015.

Although each clinic within IeDEA has its own protocol for routine follow-up, HIV-infected children who have not yet been initiated on ART are typically seen at least every 6 months.

The data abstracted for this analysis were from routine care and included region, country, site, patient demographics (sex, date of birth, date of HIV diagnosis if available, and date of enrollment in care), clinical WHO/CDC staging at enrollment and ART initiation, laboratory values and dates (CD4 cell count, CD4 percent), cotrimoxazole start date, date of ART initiation, ART regimen, and date of death, LTFU, or transfer out.

### Outcomes and key variable definitions

The outcomes of interest were (1) time to ART initiation and (2) missed opportunities for ART initiation, defined as either death or LTFU (last clinical visit >6 months before database closure and whereabouts unknown). Baseline was defined as the date of diagnosis, whether this occurred before or after enrollment in the HIV care program; when this date was unavailable, which was frequent in these contexts, we defined baseline as the date of enrollment in the HIV care program. The follow-up period was defined as the time between baseline and the date of ART initiation, death, LTFU, transfer out, or database closure (31 December 2015), whichever came first.

### Statistical analysis

We assessed the proportion of children initiating ART and the proportion with missed opportunities for ART initiation (death or LTFU) within the first 24 months after enrollment. This conservative time-point was prespecified to provide a reference analysis in scaling up HIV diagnosis and ART initiation before the treat-all era from 2015 onwards. Baseline categorical data are presented as frequency (percent), and continuous data are presented as median (interquartile range [IQR]). Continuous variables were compared using the Kruskal–Wallis test, and categorical variables using the chi-squared or Fisher’s test. Time to ART initiation was estimated using cumulative incidence functions (CIFs): mortality and LTFU were considered competing events to ART initiation [9], while those transferred out were right-censored, with the assumption that they remained in HIV care and had similar outcomes as patients still in observation.

To better understand the different patterns of ART initiation, taking into account the evolving WHO eligibility criteria for ART initiation between 2004 and 2015 [4,5,10,11], we described the percentage of children eligible for ART initiation at baseline and during follow-up according to WHO guidelines, combining clinical criteria (WHO stage 3 or 4 or AIDS, though these data were unavailable in the Southern Africa database) and severe immunodeficiency for age (CD4 ≤ 25% if age < 5 years or CD4 ≤ 350 cells/μl if age ≥ 5 years) if enrollment in care occurred prior to 1 April 2008; additional age criteria were added if enrolled later (age <1 year between 1 April 2008 and 30 June 2010, age < 2 years between 1 July 2010 and 31 May 2013, and age < 5 years on or after 1 June 2013).

Correlates of ART initiation were described in a multivariate competing risks analysis using the Fine and Gray proportional sub-distribution hazards regression model, where time of origin was baseline [12]. Explanatory variables included sex, age at baseline, region, country income as defined by the World Bank (http://data.worldbank.org/about/country-and-lending-groups), period of enrollment based on the changing WHO treatment guidelines (before April 2008, April 2008–June 2010, July 2010–May 2013, and after May 2013), and clinical/immunological criteria for ART eligibility at baseline and during follow-up. Model fit was checked graphically, plotting the Schoenfeld-type residuals against time for each of the covariates included in the model; we used the *%pshreg* SAS macro for this [13,14].

There was a high degree of variability of data collection practices across the HIV programs included in IeDEA, and some centers followed up only children who were eligible at baseline to be initiated on ART. It is likely that those programs only recorded data if children initiated ART or were intended to initiate ART before being followed up in a decentralized center. Consequently, incidence of ART initiation among all children in HIV care may be overestimated by these programs. For this reason, we performed an un-prespecified sensitivity analysis excluding the clinics where the ART coverage was >95% of all the patients followed up.

Analyses were conducted using the package *cmprsk* in R statistical software version 2.11.1 (R Foundation for Statistical Computing, Vienna, Austria). The adjusted sub-distribution hazard ratios (asHRs) were reported with their 95% confidence intervals (CIs). A *p*-value less than 0.05 was considered statistically significant.

### Ethics

Each participating IeDEA region formally agreed to contribute pediatric data, with local institutional review board and US National Institutes of Health approvals to contribute to multiregional analyses.

## Results

### Baseline characteristics and follow-up

**Fig 1:**
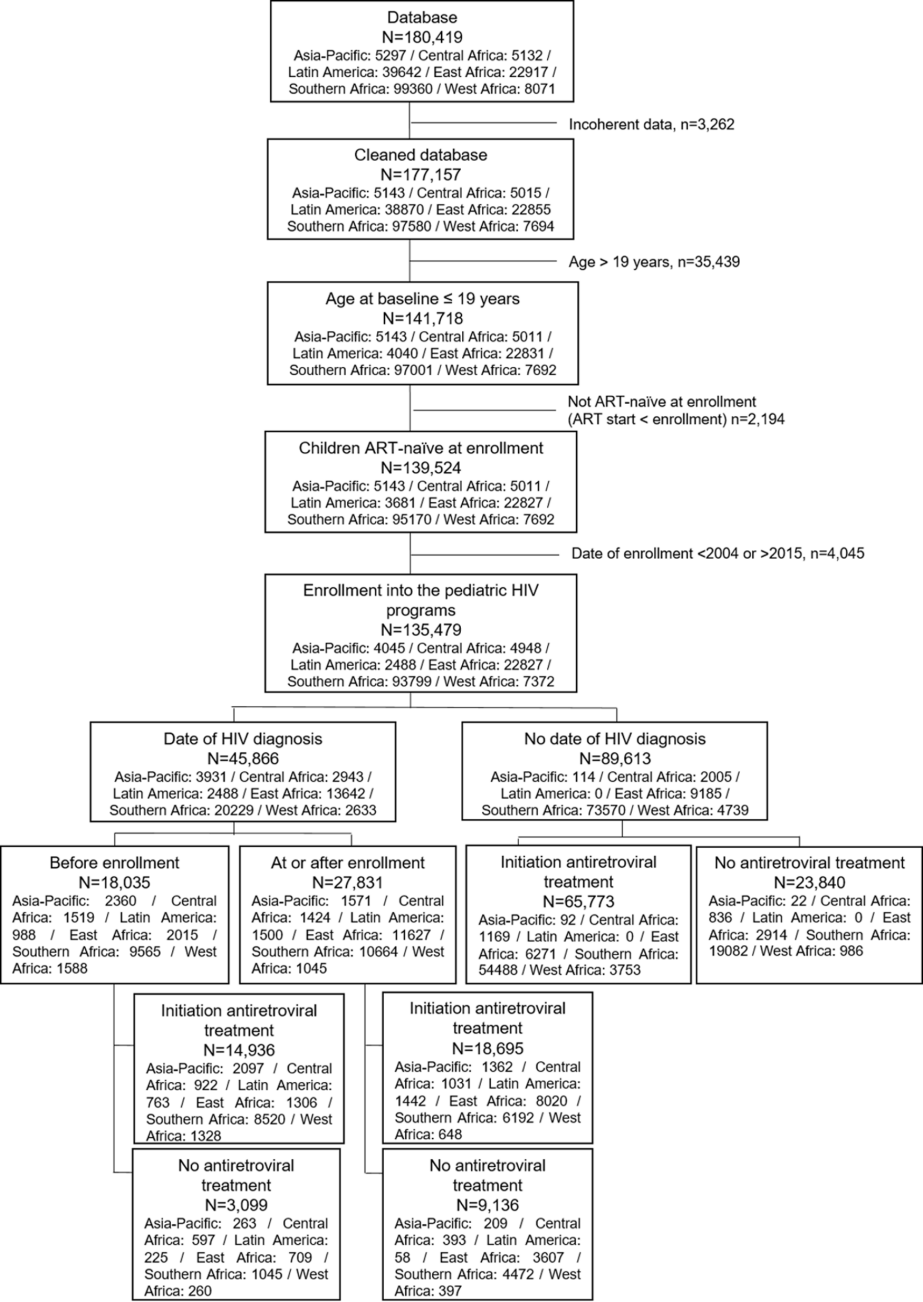
Flow diagram of the inclusion criteria of the 135,479 HIV-infected children aged 0–19 years enrolled in the IeDEA Global Cohort Consortium from 2004 to 2015.

Overall, 180,419 children and adolescents were included in the IeDEA, of whom 120,413 were <15 years; this represents about 6% of all children living with HIV worldwide according to UNAIDS estimates. Of these, 3,262 (1.9%) were excluded due to incoherent data, 35,439 (19.6%) because they were >19 years of age, 2,194 (1.2%) because they were not ART-naïve at baseline—these children most likely entered in IeDEA active files transferring from other clinics while they were already on ART but without a date of ART initiation—and 4,045 (2.2%) because the date of enrollment in the site was before 2004 or after 2015, leaving 135,479 (75.1%) who met the inclusion criteria and were included in the study; 69.2% were from Southern Africa. Among all children included, 27,831 (20.5%) had a documented date of HIV diagnosis at or after enrollment (for those HIV-exposed) and 18,035 (13.3%) had a documented date of HIV diagnosis prior to enrollment; for the remaining 89,613 (66.1%), the date of enrollment was used as baseline (Fig 1).

Table 1 describes the patients’ characteristics at baseline. Children were enrolled at a late age, 6 years in median (IQR: 2–12), but the median age varied by region, ranging from 4 years in West Africa (IQR: 2–9) and Asia-Pacific (IQR: 2–7) to 8 years in Latin America (IQR: 2–16). This variation was mainly driven by the proportion of adolescents enrolled in the HIV care programs in each region. Overall, 32.1% of children were adolescents (≥10 years) at baseline. This varied across regions, with Asia-Pacific having the smallest proportion (9.9%), and Latin America the largest (43.2%). Children aged 5–9 years were the most commonly represented group overall (23.5%). Median CD4 percentage at baseline was 17% (IQR: 10%–25%) overall: 12% in Asia-Pacific, 15% in West Africa, 16% in Central Africa and Latin America, 17% in Southern Africa, and 19% in East Africa (*p <* 0.01). At the time of their enrollment, 49.1% of children were known to be eligible for ART initiation according to WHO recommendations. This percentage ranged from 43.0% in Central Africa to 74.5% in the Asia-Pacific region. Forty-eight percent of children could not be classified for ART eligibility at baseline due to missing clinical and/or immunological data.

**Table 1:** Baseline (HIV diagnosis or, if unavailable, enrollment in care) characteristics of the 135,479 HIV-infected children included, by region, in the IeDEA Global Cohort Consortium, 2004–2015.

Characteristic	Region						Total	*p-*Value
	Asia-Pacific	Central Africa	Latin America	East Africa	Southern Africa	West Africa		
*N*	4,045	4,948	2,488	22,827	93,799	7,372	135,479	
Sex, *n* (%)								<0.01*
Males	2,082 (51.5)	2,222 (44.9)	1,106 (44.5)	9,642 (42.2)	40,486 (43.2)	3,414 (46.3)	58,952 (43.5)	
Females	1,963 (48.5)	2,726 (55.1)	1,382 (55.5)	13,185 (57.8)	53,154 (56.7)	3,480 (47.2)	75,890 (56.0)	
Unknown	0 (0.0)	0 (0.0)	0 (0.0)	0 (0.0)	159 (0.2)	478 (6.5)	637 (0.5)	
Median age at baseline (years) (IQR)	4 (2–7)	7 (3–12)	8 (2–16)	6 (3–12)	6 (2–12)	4 (2–9)	6 (2–12)	<0.01
Age at baseline, *n* (%)								<0.01
0–11 months	653 (16.1)	570 (11.5)	425 (17.1)	2,449 (10.7)	14,859 (15.8)	1,061 (14.4)	20,017 (14.8)	
12–23 months	508 (12.6)	377 (7.6)	211 (8.5)	2,014 (8.8)	11,001 (11.7)	1,114 (15.1)	15,225 (11.2)	
2–4 years	1,233 (30.5)	912 (18.4)	354 (14.2)	5,383 (23.6)	15,307 (16.3)	1,769 (24.0)	24,958 (18.4)	
5–9 years	1,249 (30.9)	1,345 (27.2)	423 (17.0)	5,798 (25.4)	21,073 (22.5)	1,904 (25.8)	31,792 (23.5)	
10–14 years	377 (9.3)	966 (19.5)	404 (16.2)	3,312 (14.5)	15,030 (16.0)	941 (12.8)	21,030 (15.5)	
15–19 years	25 (0.6)	778 (15.7)	671 (27.0)	3,871 (17.0)	16,529 (17.6)	583 (7.9)	22,457 (16.6)	
Country income, *n* (%)								<0.01^$^
Low income	593 (14.7)	4,948 (100.0)	1,704 (68.5)	22,827 (100.0)	12,671 (13.5)	2,136 (29.0)	44,879 (33.1)	
Lower middle income	2,235 (55.3)	0 (0.0)	201 (8.1)	0 (0.0)	46,275 (49.3)	5,236 (71.0)	53,947 (39.8)	
Upper middle income	1,217 (30.1)	0 (0.0)	563 (22.6)	0 (0.0)	34,853 (37.2)	0 (0.0)	36,633 (27.0)	
High income	0 (0.0)	0 (0.0)	20 (0.8)	0 (0.0)	0 (0.0)	0 (0.0)	20 (0.01)	
Year of enrollment, *n* (%)								<0.01
<April 2008	2,139 (52.9)	2,184 (44.1)	1,006 (40.4)	8,003 (35.1)	26,338 (28.1)	3,045 (41.3)	42,715 (31.5)	
April 2008–June 2010	1,009 (24.9)	1,116 (22.6)	427 (17.2)	6,437 (28.2)	23,141 (24.7)	1,429 (19.4)	33,559 (24.8)	
July 2010–May 2013	701 (17.3)	1,200 (24.3)	696 (28.0)	6,590 (28.9)	27,065 (28.9)	1,729 (23.5)	37,981 (28.0)	
≥June 2013	196 (4.8)	448 (9.1)	359 (14.4)	1,797 (7.9)	17,255 (18.4)	1,169 (15.9)	21,224 (15.7)	
Access to care, *n* (%)								<0.01
Enrolled following an HIV diagnosis	2,360 (58.3)	1,519 (30.7)	988 (39.7)	2,015 (8.8)	9,565 (10.2)	1,588 (21.5)	18,035 (13.3)	
Diagnosis at time of enrollment	918 (22.7)	1,280 (25.9)	1,171 (47.1)	9,609 (42.1)	9,459 (10.1)	552 (7.5)	22,989 (17.0)	
Diagnosis after enrollment	653 (16.1)	144 (2.9)	329 (13.2)	2,018 (8.8)	1,205 (1.3)	493 (6.7)	4,842 (3.6)	
No date of confirmed HIV diagnosis available	114 (2.8)	2,005 (40.5)	0 (0.0)	9,185 (40.2)	73,570 (78.4)	4,739 (64.3)	89,613 (66.1)	
WHO/CDC clinical stage at baseline, *n* (%)								<0.01
Stage 1/2 or CDC stage A/B	1,292 (31.9)	1,470 (29.7)	428 (17.2)	8,158 (35.7)	0 (0.0)	1,968 (26.7)	13,316 (9.8)	
Stage 3/4 or AIDS	1,925 (47.6)	1,103 (22.3)	190 (7.6)	2,137 (9.4)	0 (0.0)	2,621 (35.6)	7,976 (5.9)	
Unknown	828 (20.5)	2,375 (48.0)	1,870 (75.2)	12,532 (54.9)	93,799 (100.0)	2,783 (37.8)	114,187 (84.3)	
Severe immunodeficiency for age at baseline**, *n* (%)								<0.01
Yes	2,177 (53.8)	948 (19.2)	787 (31.6)	6,610 (29.0)	32,314 (34.5)	2,996 (40.6)	45,832 (33.8)	
No	772 (19.1)	954 (19.3)	555 (22.3)	5,855 (25.6)	19,903 (21.2)	1,320 (17.9)	29,359 (21.7)	
Missing	1,096 (27.1)	3,046 (61.6)	1,146 (46.1)	10,362 (45.4)	41,582 (44.3)	3,056 (41.5)	60,288 (44.5)	
Clinical^£^ or immunological** ART eligibility at baseline, *n* (%)								<0.01
Yes	2,864 (70.8)	1,676 (33.9)	893 (35.9)	8,082 (35.4)	32,314 (34.5)	4,416 (59.9)	50,245 (37.1)	
No	370 (9.1)	462 (9.3)	174 (7.0)	2,413 (10.6)	0 (0.0)	430 (5.8)	3,849 (2.8)	
Missing	811 (20.0)	2,810 (56.8)	1,421 (57.1)	12,332 (54.0)	61,485 (65.5)	2,526 (34.3)	81,385 (60.1)	
Eligible for ART according to WHO recommendations^&^ at baseline, *n* (%)								<0.01
Yes	3,014 (74.5)	2,140 (43.2)	1,222 (49.1)	10,479 (45.9)	44,632 (47.6)	4,995 (67.8)	66,482 (49.1)	
No	300 (7.4)	459 (9.3)	156 (6.3)	2,379 (10.4)	0 (0.0)	380 (5.2)	3,674 (2.7)	
Missing	731 (18.1)	2,349 (47.5)	1,110 (44.6)	9,969 (43.7)	49,167 (52.4)	1,997 (27.1)	65,323 (48.2)	
Measure of CD4 cell count available, *n* (%)	2,976 (73.6)	1,562 (31.6)	1,658 (66.6)	13,506 (59.2)	54,353 (57.9)	4,839 (65.6)	78,894 (58.2)	
Median CD4 cell count (cells/μl) (IQR)	353 (63–830)	427 (217–668)	395 (182–718)	484 (232–820)	418 (207–758)	453 (163–831)	430 (203–773)	<0.01
Measure of CD4 percentage available, *n* (%)	2,917 (72.1)	432 (8.7)	526 (21.1)	9,479 (41.5)	42,534 (45.3)	3,548 (48.1)	59,436 (43.9)	
Median CD4 percentage (IQR)	12 (4–22)	16 (11–23)	16 (8–26)	19 (11–27)	17 (11–25)	15 (7–22)	17 (10–25)	<0.01

*Males versus others.

^$^Low/lower middle/unknown versus upper middle/high.

**Severe immunodeficiency for age: CD4 ≤ 25% if age < 5 years or CD4 ≤ 350 cells/μl if age ≥ 5 years.

^£^Clinical stage WHO 3 or 4 or AIDS.

^&^Baseline before 1 April 2008: clinical or immunological eligibility; baseline 1 April 2008–30 June 2010: clinical or immunological eligibility or children <1 year; baseline 1 July 2010–31 May 2013: clinical or immunological eligibility or children <2 years; baseline on or after 1 June 2013: clinical or immunological eligibility or children <5 years.

CDC, Centers for Disease Control and Prevention.

Access to care varied greatly over regions. Overall, 31.5% of children were enrolled prior to April 2008; this represented 52.9% of the cohort in the Asia-Pacific region but only 28.1% in Southern Africa. Of all children in the database, 66.1% did not have a date of confirmed HIV diagnosis in the database: in Latin America and Asia-Pacific, a smaller proportion of children lacked HIV diagnosis date (0.0% and 2.8%, respectively), but in Southern Africa this proportion was 78.4%. Among those with a date of confirmed HIV diagnosis, 39.3% of children were diagnosed prior to enrollment in IeDEA, ranging from 14.8% in East Africa to 60.3% in West Africa (*p <* 0.001; Table 1); the overall median time to enrollment in care since HIV diagnosis among these children was 1 month (IQR: 0–7).

### Incidence of ART initiation

Among the 135,479 children included in the study, 99,404 (73.4%) initiated ART. The median time to ART initiation was 1 month (IQR: 0–6 months); 1.9% died, 1.4% were transferred out, and 20.4% were lost to follow-up before any ART initiation (Table 2). Time to ART initiation varied according to the timing of diagnosis. Among those diagnosed prior to enrollment in care, the median time to ART since diagnosis was 4 months (IQR: 1–19); among those diagnosed after enrollment in care, median time to ART was 3 months (IQR: 1–10). Among those with no documented date of HIV diagnosis, median time from enrollment to ART was 1 month (IQR: 0–5). Finally, among those enrolled at time of diagnosis, the median time from baseline to ART was 2 months (IQR: 0–8).

**Table 2:** Outcomes among the 135,479 pre-ART children in the IeDEA Global Cohort Consortium, 2004–2015.

Variable or outcome	Region						Total	*p-*Value
	Asia-Pacific	Central Africa	Latin America	East Africa	Southern Africa	West Africa		
*N*	4,045	4,948	2,488	22,827	93,799	7,372	135,479	
Death before ART initiation, *n* (%)	76 (1.9)	101 (2.0)	20 (0.8)	730 (3.2)	1,397 (1.5)	218 (3.0)	2,542 (1.9)	<0.01
Transferred out before ART initiation, *n* (%)	222 (5.5)	194 (3.9)	10 (0.4)	322 (1.4)	939 (1.0)	220 (3.0)	1,907 (1.4)	<0.01
Loss to follow-up (>6 months) before ART initiation, *n* (%)	94 (2.3)	1,353 (27.3)	197 (7.9)	5,116 (22.4)	19,805 (21.1)	1,047 (14.2)	27,612 (20.4)	<0.01
Censored due to end of study, *n* (%)	102 (2.5)	178 (3.6)	56 (2.3)	1,062 (4.7)	2,458 (2.6)	158 (2.1)	4,014 (3.0)	
ART initiation, *n* (%)	3,551 (87.8)	3,122 (63.1)	2,205 (88.6)	15,597 (68.3)	69,200 (73.8)	5,729 (77.7)	99,404 (73.4)	<0.01
On ART at baseline, *n* (%)	132 (3.7)	157 (5.0)	74 (3.4)	1,562 (10.0)	18,964 (27.4)	913 (15.9)	21,802 (21.9)	<0.01
Median duration between baseline and ART initiation or last contact (months) (IQR)	2 (1–12)	5 (1–26)	4 (1–24)	3 (1–11)	1 (0–5)	1 (0–6)	1 (0–7)	<0.01
Median duration between ART eligibility and ART initiation/last contact (months) (IQR)	2 (1–10)	4 (1–23)	4 (1–22)	3 (1–11)	1 (0–5)	1 (0–5)	1 (0–6)	<0.01
Closure date	30 May 2015	21 Apr 2016	30 May 2016	31 Dec 2014	29 Jan 2016	17 May 2016		

**Fig 2:**
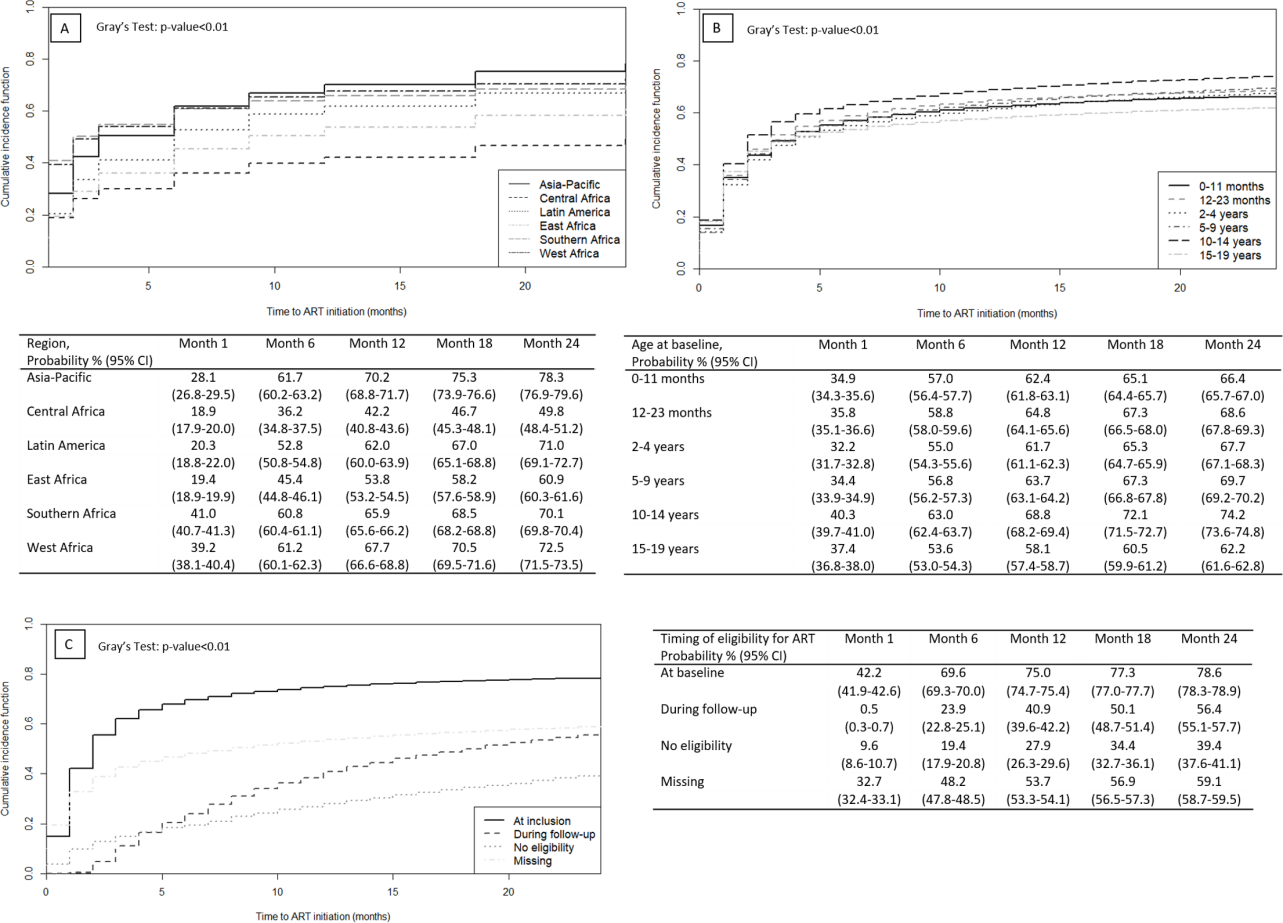
Cumulative incidence functions for ART initiation by region, age at baseline, and timing of eligibility for ART, among the 135,479 HIV-infected pre-ART children within the IeDEA Global Cohort Consortium, 2004–2015. By region (A), age at baseline (B), and timing of eligibility for ART (C).

After 1 month of pre-ART follow-up, the CIF for ART initiation in all HIV-infected children was estimated to be 35.7% (95% CI: 35.4%–35.9%) while the CIF for missed opportunities (death or LTFU) was 10.7% (95% CI: 10.5%–10.9%). By 24 months of pre-ART follow-up, the CIF for ART initiation had reached 68.2% (95% CI: 67.9%–68.4%), and the 24-month cumulative incidence for missed opportunities was 19.3% (95% CI: 19.1%–19.5%). Fig 2A presents the 24-month CIF for time to ART initiation since baseline by region. It was 71.0% (95% CI: 69.1%–72.7%) and 78.3% (95% CI: 76.9%–79.6%) for Latin America and the Asia-Pacific, respectively. In sub-Saharan Africa, the 24-month CIF for ART initiation was significantly lower, ranging from 49.8% (95% CI: 48.4%–51.2%) in Central Africa to 60.9% (95% CI: 60.3%–61.6%) in East Africa, 70.1% (95% CI: 69.8%–70.4%) in Southern Africa, and 72.5% (95% CI: 71.5%–73.5%) in West Africa. We also noted that results differed by age at baseline: children and adolescents aged 15–19 years and those aged <1 year at baseline had the lowest ART initiation rates compared to other ages, with a 24-month CIF of 62.2% (95% CI: 61.6%–62.8%) and 66.4% (95% CI: 65.7%–67.0%), respectively (Fig 2B).

Fig 2C presents CIFs for ART initiation according to ART eligibility at baseline. Among children eligible at baseline, the 24-month CIF was 78.6% (95% CI: 78.3%–78.9%) compared to 56.4% (95% CI: 55.1%–57.7%) among those known not to be eligible at baseline who became eligible during follow-up. We also note that the CIF for ART initiation reached 39.4% (95% CI: 37.6%–41.1%) at 24 months among children who did not meet ART eligibility criteria. Among those with unknown eligibility criteria at baseline, the 24-month CIF for ART initiation was 58.7% (95% CI: 58.3%–59.1%) (S3 Fig).

### ART initiation according to ART eligibility

**Fig 3:**
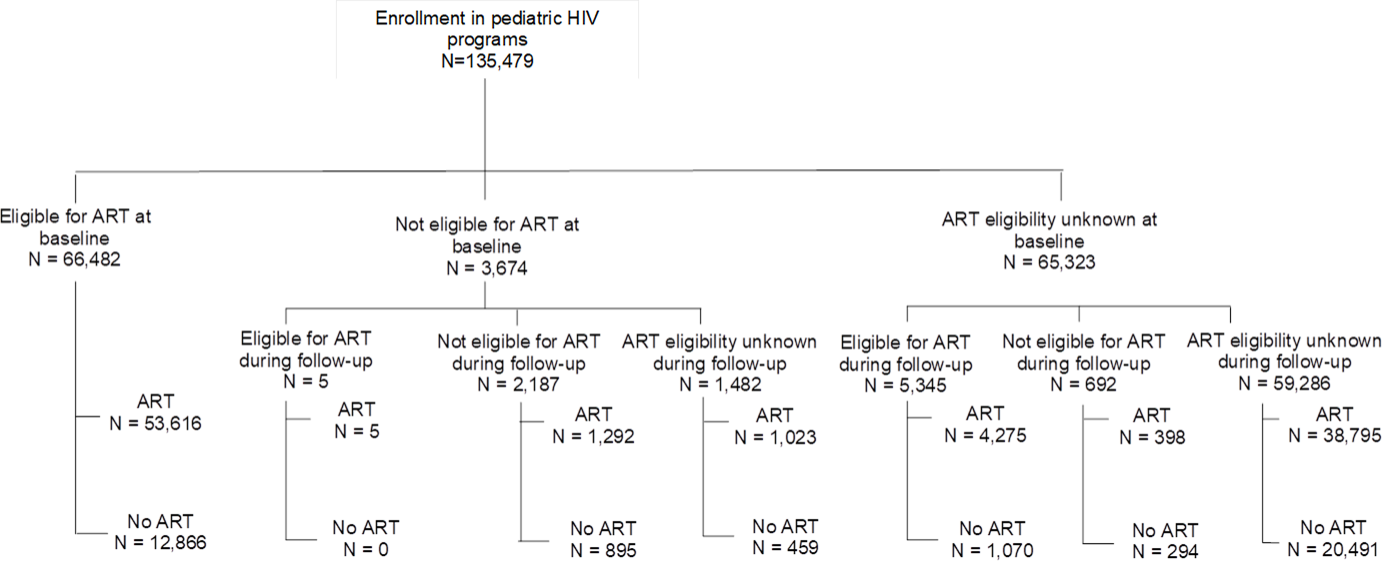
ART initiation according to ART eligibility at baseline and during follow-up among the 135,479 HIV-infected children in the IeDEA Global Cohort Consortium, 2004–2015.

To better understand the patterns of ART initiation, we present the ART eligibility for the overall population (Fig 3). Of the 135,479 children included in the analysis, 66,482 (49.1%) were known to be eligible for ART initiation according to WHO criteria at baseline, of whom 53,616 (80.6%) initiated ART. Among the 3,674 (2.7%) not eligible for ART at baseline, 5 (0.1%) became eligible during follow-up, of whom all initiated ART. Among the 65,323 (48.2%) who were not classified for ART eligibility at baseline due to missing clinical and/or immunological data, 5,345 (8.2%) became eligible, of whom 4,275 (80.0%) initiated ART, and 692 (1%) did not meet eligibility criteria during follow-up, of whom 398 (57%) initiated ART. Overall, 13,936 (19.4%) children known to be eligible for ART initiation, either at baseline or during follow-up, never accessed treatment over the first 24 months of follow-up. These missed opportunities for ART initiation highlight a substantial proportion of unmet needs.

**Fig 4:**
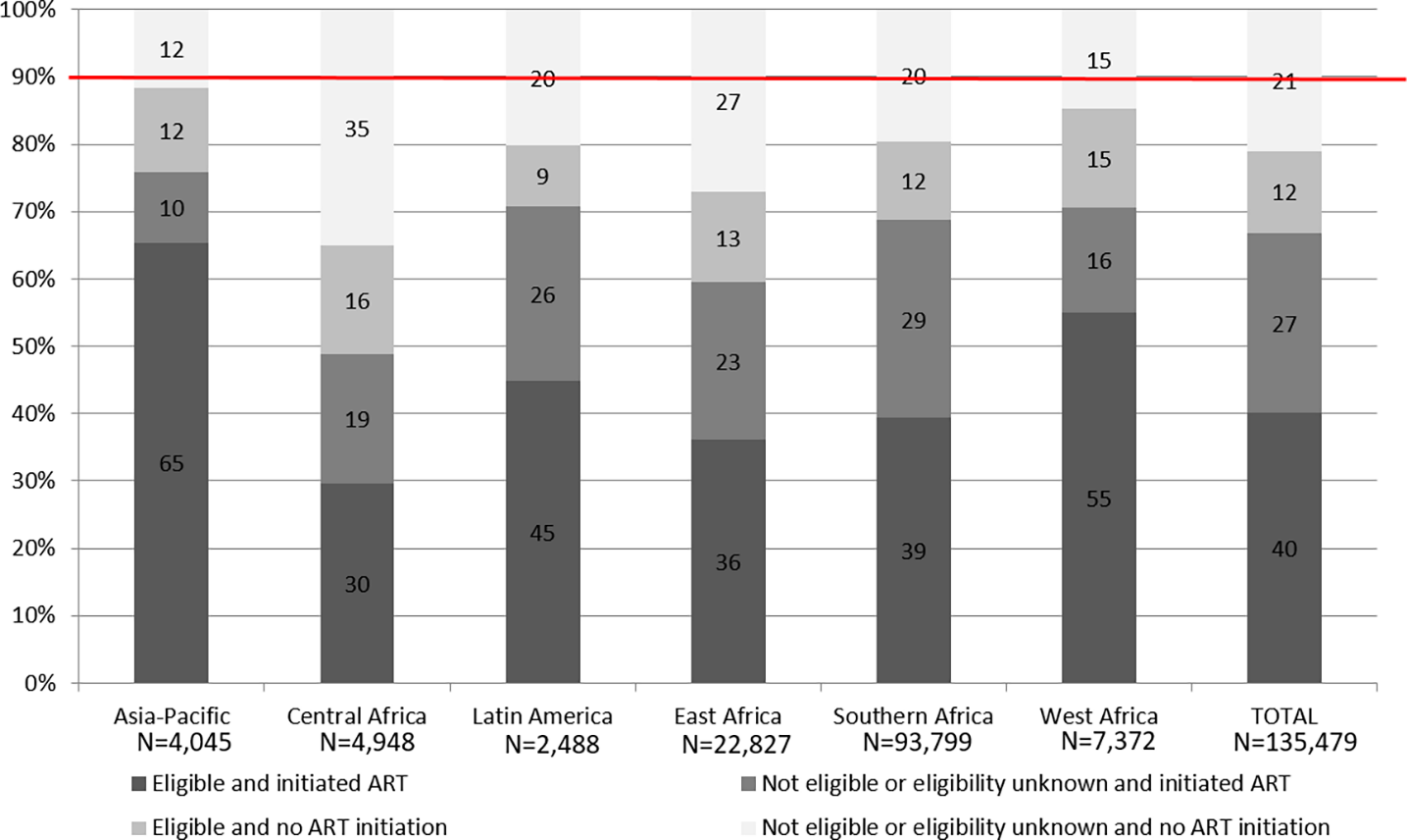
ART initiation after 24 months of follow-up according to eligibility at last contact, by region, among the 135,479 HIV-infected children (including those lost to follow-up or deceased prior to ART initiation) in the IeDEA Global Cohort Consortium, 2004–2015.

In 2015, no region had yet reached the UNAIDS target of 90% of those diagnosed with HIV infection on ART, though the Asia-Pacific, Latin America, and Southern and West Africa IeDEA regions had percentages of ART initiation close to 80% (Fig 4). Central and East Africa had the lowest coverage (49% and 59%, respectively). A substantial proportion of eligible children did not initiate ART during the study period, ranging from 9% in Latin America to 15% in West Africa and 16% in Central Africa.

### Correlates of ART initiation

In the multivariate Fine and Gray analysis, sex, region, age at baseline, period of enrollment, country income, and clinical or immunological eligibility were all associated with ART initiation (Table 3). Adjusted for the above variables, females were less likely to initiate ART compared to their male counterparts (asHR: 0.94, 95% CI: 0.92–0.95). Compared to Latin America, 3 sub-Saharan African regions were less likely to initiate ART, with asHRs ranging from 0.69 (95% CI: 0.66–0.72) in Central Africa and 0.80 (95% CI: 0.77–0.83) in East Africa to 0.93 (95% CI: 0.90–0.97) in Southern Africa. Children from the Asia-Pacific and West Africa regions had similar hazard of treatment initiation compared to those from Latin America (asHR: 1.01, 95% CI: 0.97–1.05, and asHR: 1.02, 95% CI: 0.97–1.06, respectively). Adolescents aged 10–14 years at baseline were the most likely to initiate ART compared to all other age groups. Infants aged <1 year and adolescents aged ≥15 years were less likely to initiate ART compared to those 10–14 years old (asHR: 0.83, 95% CI: 0.81–0.85, and asHR: 0.73, 95% CI: 0.71–0.75, respectively). Children enrolled prior to June 2013 were also less likely to initiate ART than those enrolled more recently, adjusted for other variables, and we noted a tendency towards lower likelihood of initiating ART in earlier enrollment periods (asHR: 0.78, 95% CI: 0.76–0.80, for those with enrollment in July 2010–May 2013; asHR: 0.63,95% CI: 0.61–0.64, for those enrolled in the period April 2008–June 2010; and asHR: 0.57, 95% CI: 0.55–0.58, for those enrolled prior to April 2008) compared to those enrolled in or beyond June 2013. ART initiation was also associated with country income: children from countries of low or lower middle income were less likely to initiate ART compared to those from upper-middle- and high-income settings (asHR: 0.76, 95% CI: 0.75–0.78). Finally, we found that children who became eligible for ART initiation (per clinical and/or immunological criteria, but not on age criteria—that was a separate variable) during follow-up were less likely to initiate ART compared to those who were eligible at baseline (asHR: 0.61, 95% CI: 0.60–0.63).

**Table 3:** Factors associated with ART initiation during pre-ART follow-up in HIV-infected children (*n =* 135,479) in the IeDEA Global Cohort Consortium, 2004–2015.

Characteristic	Univariate analysis			Full model		
	sHR	95% CI	*p-*Value	asHR	95% CI	*p-*Value
Sex			<0.01			<0.01
Males	1	—		1	—	
Females	0.90	0.89–0.91		0.94	0.92–0.95	
Missing	0.73	0.65–0.82		0.58	0.51–0.65	
Region			<0.01			<0.01
Latin America	1	—		1	—	
Asia-Pacific	1.14	1.10–1.18		1.01	0.97–1.05	
Central Africa	0.61	0.59–0.64		0.69	0.66–0.72	
East Africa	0.73	0.70–0.75		0.80	0.77–0.83	
Southern Africa	0.99	0.96–1.02		0.93	0.90–0.97	
West Africa	1.05	1.01–1.09		1.02	0.97–1.06	
Age at baseline			<0.01			<0.01
0–11 months	0.79	0.77–0.80		0.83	0.81–0.85	
12–23 months	0.84	0.82–0.86		0.86	0.84–0.88	
2–4 years	0.84	0.82–0.86		0.88	0.86–0.90	
5–9 years	0.89	0.87–0.91		0.97	0.95–0.98	
10–14 years	1	—		1	—	
15–19 years	0.74	0.72–0.75		0.73	0.71–0.75	
Period of enrollment			<0.01			<0.01
<April 2008	0.64	0.63–0.65		0.57	0.55–0.58	
April 2008–June 2010	0.67	0.66–0.69		0.63	0.61–0.64	
July 2010–May 2013	0.80	0.78–0.82		0.78	0.76–0.80	
≥June 2013	1	—		1	—	
Country income			<0.01			<0.01
Upper middle/high	1	—		1	—	
Low/lower middle	0.73	0.72–0.74		0.76	0.75–0.78	
Clinical* or immunological** eligibility			<0.01			<0.01
At baseline	1	—		1	—	
During follow-up	0.56	0.55–0.58		0.61	0.60–0.63	
Never	0.40	0.39–0.42		0.47	0.45–0.48	
Missing	0.63	0.62–0.64		0.61	0.60–0.62	

Reference groups were chosen based on those with the highest likelihood for ART initiation.

*WHO clinical stage 3/4 or AIDS.

**Severe immunodeficiency for age: CD4 ≤ 25% if age < 5 years or CD4 ≤ 350 cells/μl if age ≥ 5 years.

asHR, adjusted sub-distribution hazard ratio; sHR, sub-distribution hazard ratio.

### Sensitivity analysis

Overall, 112,134 children were followed up in clinics where ART coverage was ≤95%. Results are presented in the Supporting Information (S2 and S3 Figs; S1 Table). Among these children, the 24-month CIF for ART initiation was estimated to be much lower than in the whole population, at 62.9% (95% CI: 62.6%–63.2%), and the 24-month probability for missed opportunities for ART was higher, at 23.3% (95% CI: 23.1%–23.6%), than in the whole population (S2 Fig). The children from Asia-Pacific had the highest ART initiation rates, reaching 73.1% (95% CI: 71.4%–74.8%), compared to all others, including those from sub-Saharan Africa, with 49.8% (95% CI: 48.4%–51.2%) in Central Africa, 60.2% (95% CI: 58.8%–61.7%) in West Africa, 60.9% (95% CI: 58.8%–61.7%) in East Africa, and 60.2% (95% CI: 58.8%–61.7%) in Southern Africa (S3 Fig). In the multivariate Fine and Gray competing risk analysis, we did not observe significant changes in correlates of ART initiation in this population, except for the effect of region, with the lowest asHR for ART initiation in sub-Saharan Africa compared to Latin America, ranging from 0.60 in Central Africa (95% CI: 0.57–0.63) and West Africa (95% CI: 0.57–0.63) and 0.70 in East Africa (95% CI: 0.67–0.73) to 0.80 in Southern Africa (95% CI: 0.77–0.83) (S1 Table).

## Discussion

This pooled analysis from the IeDEA Global Cohort Consortium documents time to ART initiation since enrollment in HIV programs treating HIV-infected children and adolescents between the ages of 0 and 19 years within multiple geographic regions, between 2004 and 2015. We report 3 major findings. First, in HIV-infected children and adolescents, the cumulative incidence of initiating ART within 24 months of enrollment into a program or HIV diagnosis was 68%, with a substantial risk for mortality or LTFU before ART initiation (19%), representing multiple missed opportunities for ART initiation. Second, among children eligible for ART initiation and followed up, 19% did not initiate treatment within the first 24 months of follow-up. Third, we report a number of inequities in ART access: female sex, children <10 years at baseline (and those <1 year in particular), adolescents aged 15–19 years at baseline (compared to those aged 10–14 years), those becoming eligible during follow-up (compared to those eligible at baseline), and those living in sub-Saharan Africa compared to other regions were all less likely to initiate treatment.

The 24-month cumulative incidence for ART initiation was 68%. According to ART eligibility, this was 78.6% among those eligible at baseline and 56.4% among those who became eligible during the study. While these rates are low compared to the UNAIDS target of 90% of those diagnosed with HIV, they are encouraging compared to previous available studies. For example, in South Africa, only 34.8% of ART-eligible HIV-infected children initiated ART in 2003 [15]. More recently, in Lesotho, 41.2% of eligible children initiated ART in 2008 [16]. In Côte d’Ivoire, 55% of eligible children initiated ART in 2009 [17]. Still, the rate of ART initiation among children often lags compared to adults and occurs late, despite progressive guidelines stressing immediate treatment initiation for the youngest children regardless of immunological/clinical status since 2008 [18].

At inclusion in HIV programs, 66.1% of children did not have a date of confirmed HIV diagnosis, median age was 6 years, and 49.1% were already eligible for ART, highlighting the late access to ART. Delayed ART initiation is most likely the result of late access to HIV diagnosis for HIV-exposed children [19,20]. Difficulties in identifying HIV-exposed infants, limited capacity to perform routine virological testing in HIV-exposed infants, and long result turnaround time remain important barriers to timely initiation of treatment [20]. In addition, the lack of integration of prevention of mother-to-child transmission and pediatric HIV care programs hampers the delivery of early infant diagnosis [21]. This is mainly related to infrastructure limitations and time constraints as well as staff shortages [20,22,23]. In older children, similar structural barriers have been identified [21], along with additional key barriers such as stigma, lack of knowledge in the adolescent population, and socio-cultural beliefs [24–27].

There are many points at which children may drop out the HIV care cascade. First and foremost, linkage to care after HIV diagnosis remains a complex issue [28]. There are many reasons for failing to link a child to care including fear of stigma [29–31], community and economic factors such as lack of support and finances for transport, missed days of work, and healthcare worker and infrastructure constraints (e.g., drug stock outs, lack of knowledge among providers on when to prescribe ART, patients missing appointments, eligible patients not identified appropriately) [32,33]. Challenges continue even after linkage to care. Although our results demonstrate the feasibility of large-scale ART rollout for children, 1.9% died and 20.4% were lost to follow-up before ART initiation. Some of the children who were lost to HIV care programs may represent undocumented mortality, but they may also be out of care or may have transferred to other facilities without documentation. Once children were linked to HIV care, we also observed suboptimal rates of ART initiation among children who became eligible during follow-up compared to those eligible at enrollment, further undermining the HIV care cascade.

Direct comparisons with other studies are made difficult by differences in methodology and definition of outcomes, but our observations underline the many missed opportunities for ART initiation and the difficulties in keeping children in the pre-ART care cascade. Low retention among HIV-diagnosed patients waiting to initiate ART, as observed in our study, has also been previously described in both adult and pediatric populations. Most losses happen between HIV diagnosis and CD4 staging [34]. Current guidelines no longer require CD4 staging to start ART, which may contribute to improved retention in care and access to ART.

Weaknesses in the continuity of care services must urgently be addressed in order to improve ART coverage and survival among HIV-infected children. For perinatally infected children, other interventions such as family-centered models have also been proposed to improve linkage to care after diagnosis [35,36]. In older children, use of youth-friendly models of care may be an important intervention [37–39]. Regardless of the mode and age at infection, multiple efforts are necessary to reach high uptake of services [40,41]. HIV testing and care need to be decentralized and brought to communities [42]. There is a need to support families and healthcare workers to provide HIV services for children [6,43,44]. Finally, the “test and treat” strategy recommended by WHO in 2015 that advocates starting HIV-infected individuals on ART immediately regardless of any eligibility criteria could further prevent these missed opportunities for ART initiation among HIV-diagnosed children.

We observed disparities in ART initiation between regions, with 24-month cumulative incidence of ART initiation ranging from <75% in sub-Saharan Africa to 78.3% in Asia-Pacific. This variability could be partly explained by the smaller number of HIV-infected children treated in Asia-Pacific compared to sub-Saharan Africa, with a larger sample size [6].

We also highlight other inequities in the rollout of ART. Females, children aged <10 years, and in particular those aged <1 year, along with adolescents aged ≥15 years, were less likely to initiate treatment. Previous studies have reported on missed opportunities for ART initiation in both very young children, mostly explained by early mortality before accessing HIV diagnosis and subsequent ART initiation, and adolescents, where fear of stigma in the family and community as well as parental consent requirements are major barriers [25,40,45]. Missed opportunities for ART initiation in adolescents aged 15–19 years could also be a reflection of noncompliance with visits. In addition, we observed that children who became eligible during follow-up were less likely to initiate treatment (asHR: 0.69, 95% CI: 0.68–0.70) compared to those eligible at enrollment. From a programmatic point of view, this observation strongly supports the universal “test and treat” strategy [6].

Our results indicate insufficient levels of ART initiation among children who were treatment eligible over the whole study period, but we also observed a gradual improvement in these rates during more recent time periods. As WHO guidelines began recommending universal ART in all children and adolescents irrespective of clinical stage or CD4 count in 2015, we expect the number needed to be treated increased in 2016. While there are still many obstacles that will impede the target of 90% ART coverage, there is an ethical priority to trace all HIV-exposed children in order to determine HIV status and link them into care if HIV infected and to treat all children who have already linked to care.

This study has major strengths but also several limitations. First, time to ART initiation since HIV diagnosis may be incorrectly estimated as data regarding confirmed dates of HIV diagnosis were scarce. We used enrollment as a proxy for HIV diagnosis, and therefore time between HIV diagnosis and ART initiation is likely underestimated in programs that do not have well-documented HIV diagnosis dates. Furthermore, a left-truncation phenomenon would mask deaths among HIV-infected children between their HIV diagnosis and inclusion into HIV programs. This survivor bias undoubtedly leads to further underestimation of the incidence of missed opportunities for ART initiation among HIV-infected children, and, consequently, the true cumulative incidence of ART initiation among all HIV-infected children is likely lower than that estimated by these data. Our results illustrate this well: programs where higher proportions were eligible at diagnosis seemed to do better in terms of ART initiation, whereas in reality these programs were doing worse as many children were diagnosed too late, with advanced HIV disease at entry. Imputation of time of diagnosis for those with missing date of diagnosis could have addressed this limitation in theory, but missing values were too numerous for this to be done. Because diagnosis is often performed at the same time as inclusion in care, our results reflect as best as possible the situation in routine care.

Second, we observed limitations inherent to data quality: 45% of children had missing data on variables used to assess ART eligibility. We thus advise caution in the interpretation of our results, particularly in the context of current universal treatment recommendations. Third, 24-month incidence of ART initiation and missed opportunities for care were derived from data collected over a 10-year period, during which both national and international guidelines varied over time. Although we adjusted for evolving ART eligibility criteria during follow-up in our final model to address this, further analyses would be necessary to better describe the progress made and treatment gaps on a national level.

Fourth, the outcomes of children lost to follow-up were not well known, and the high proportion of children lost to follow-up (20.4%) includes undocumented mortality, those out of care, and silent transfers. Both death and being out of care represent poor outcomes from missed opportunities, but silent transfers may or may not represent ultimate ART initiation. In the absence of outreach data, it is unclear how these results should be interpreted. To overcome these limitations, we combined mortality and LTFU as a single outcome (of programmatic failure) in estimating the cumulative rate of missed opportunities for ART initiation after enrollment in an HIV care program.

Fifth, HIV programs across and within regions vary. Some programs were specific to children once they started therapy, making it likely that those programs only recorded data if children initiated ART or were intended to initiate ART but not during the pre-ART period. As a result, our analysis may further overestimate the overall proportion of HIV-infected children starting ART as the denominator of all children may have shrunk in these instances. In sensitivity analyses where we excluded such programs from consideration, we observed no significant differences in our results, except in overall and regional ART initiation probabilities, which, as expected, were lower when restricted to sites with ART coverage ≤95%.

The mitigating factor of all these limitations is that the bias in estimating overall cumulative ART initiation rates goes in one direction, leading to overestimation of the cumulative rate of the start of treatment. This actually strengthens rather than undermines our conclusions regarding the continued challenge of universal care and treatment of children and adolescents living with HIV around the world. In addition, our study is the largest study reported to our knowledge to document the global pre-ART cascade in pediatrics in 2015, including data from a large number of diverse programs with significant geographic coverage; this study is a generalizable, authoritative investigation of the state of the worldwide response to the HIV epidemic in pediatric and adolescent populations living with HIV.

In conclusion, this large global cohort study of children with HIV reported a 24-month cumulative incidence of ART initiation of 68.2% between 2004 and 2015, and a high 19.3% cumulative incidence of program attrition prior to treatment start driven by mortality and loss to programs. Given the limitations to our study data, actual coverage of ART initiation in children during the study period is likely to have been lower than the estimates reported. As of 2015, there remain many obstacles to ART initiation, with substantial risks of loss to programs and death before ART initiation in the context of incomplete early infant diagnosis, linkage to care, and treatment initiation even after enrollment in care. In particular, infants <1 year of age and older adolescents urgently need more effective and targeted interventions to improve their HIV testing uptake and access to ART in order to facilitate their survival. With expanding adoption of universal treatment recommendations since 2015, it will be crucial to further monitor progress and identify gaps in ART coverage to achieve the 90-90-90 targets for children and adolescents.

## Supporting information

S1 STROBE Checklist (DOC)

S1 Fig Estimated cumulative incidence functions (CIFs) for ART initiation and death/loss to follow-up as competing events in 112,134 HIV-infected children. Pediatric IeDEA Global Cohort Consortium, 2004–2015.
(TIF)

S2 Fig Cumulative incidence functions (CIFs) for ART initiation by region among 112,134 HIV-infected children. IeDEA Global Cohort Consortium, 2004–2015.
(TIF)

S3 Fig Estimated cumulative incidence functions (CIFs) for ART initiation and death/loss to follow-up as competing events in the 65,323 HIV-infected children with ART eligibility unknown at baseline. IeDEA Global Cohort Consortium, 2004–2015.
(TIF)

S1 Table Factors associated with ART initiation during pre-ART follow-up in HIV-infected children (*n =* 112,134). IeDEA Global Cohort Consortium, 2004–2015.
(DOCX)

S1 Text Membership of the IeDEA Pediatric Working Group and IeDEA global funding acknowledgments. (DOCX)

S1 Concept Plan (PDF)

## Abbreviations

ART: antiretroviral therapy
asHR: adjusted sub-distribution hazard ratio
CI: confidence interva
CIF: cumulative incidence function
IeDEA: International Epidemiology Databases to Evaluate AIDS
IQR: interquartile range
LTFU: loss to follow-up
UNAIDS: Joint United Nations Programme on HIV/AIDS
WHO: World Health Organization
