# Supplementary material for: Access to antiretroviral therapy in HIV-infected children aged 0–19 years in the International Epidemiology Databases to Evaluate AIDS (IeDEA) Global Cohort Consortium, 2004–2015: A prospective cohort study
Source: PLoS Med. 2018 May 4;15(5):e1002565. doi: 10.1371/journal.pmed.1002565 (PMC5935422; doi:10.1371/journal.pmed.1002565)
Supplement: S1 STROBE Checklist — (DOC) [file pmed.1002565.s001.doc]

S1 STROBE Checklist

STROBE Statement—Checklist of items that should be included in reports of ***cohort studies***

|  | Item No | Recommendation |
| --- | --- | --- |
| **Title and abstract** | 1 | (*a*) Indicate the study’s design with a commonly used term in the title or the abstract  Title: “Access to Antiretroviral Therapy in HIV-Infected Children Aged 0-19 Years in the International Epidemiology Databases to Evaluate AIDS Global Cohort Consortium, 2004- 2015: a prospective cohort study.” |
|  |  | (*b*) Provide in the abstract an informative and balanced summary of what was done and what was found  Provided in abstract ‘Methods and Findings’ section. |
| Introduction | | |
| Background/rationale | 2 | Explain the scientific background and rationale for the investigation being reported  Explained in Introduction paragraphs 1-3 |
| Objectives | 3 | State specific objectives, including any prespecified hypotheses  Stated in Introduction paragraph 4 (last sentence) |
| Methods | | |
| Study design | 4 | Present key elements of study design early in the paper  Presented in Methods paragraph 1 and first sentence of paragraph 2. |
| Setting | 5 | Describe the setting, locations, and relevant dates, including periods of recruitment, exposure, follow-up, and data collection  Described in Introduction last paragraph and Methods paragraph 1 |
| Participants | 6 | (*a*) Give the eligibility criteria, and the sources and methods of selection of participants. Describe methods of follow-up  Described in Methods paragraph 1 |
|  |  | (*b*) For matched studies, give matching criteria and number of exposed and unexposed  Not a matched study |
| Variables | 7 | Clearly define all outcomes, exposures, predictors, potential confounders, and effect modifiers. Give diagnostic criteria, if applicable  Defined in Methods paragraphs 2 -5. |
| Data sources/ measurement | 8* | For each variable of interest, give sources of data and details of methods of assessment (measurement). Describe comparability of assessment methods if there is more than one group  Described in Methods paragraph 3-4 |
| Bias | 9 | Describe any efforts to address potential sources of bias  Sensitivity analyses described in Methods paragraph 6. |
| Study size | 10 | Explain how the study size was arrived at  There was no a priori sample size calculation. The intention of this project was to get as large and globally representative sample of children and adolescents enrolling in an HIV vare program waiting to initiate ART as possible. |
| Quantitative variables | 11 | Explain how quantitative variables were handled in the analyses. If applicable, describe which groupings were chosen and why  This is partially explained in the methods section paragraph 4 and 5. Additionally, from presentation of the results in Tables 1, 3, S1 it can be seen that quantitative variables (age and year of enrolment) were presented and analysed in descriptive as well as multivariable analysis as categorical variables. |
| Statistical methods | 12 | (*a*) Describe all statistical methods, including those used to control for confounding  Statistical methods are detailed in Methods paragraphs 3 and 5-6. |
|  |  | (*b*) Describe any methods used to examine subgroups and interactions  Methods to check model fit are described in paragraph 5. Subgroup analyses was a specific sensitivity analysis described in Methods paragraph 6. |
|  |  | (*c*) Explain how missing data were addressed  Handing of missing data is described in Methods paragraph 2 and further discussed in Discussion, end of paragraph 5 |
|  |  | (*d*) If applicable, explain how loss to follow-up was addressed  LTFU was a competing even accounted for in out modelling strategy: methods, paragraph 3 |
|  |  | (*e*) Describe any sensitivity analyses  Sensitivity analyses are described in Methods paragraph 6. |
| Results | | |
| Participants | 13* | (a) Report numbers of individuals at each stage of study—eg numbers potentially eligible, examined for eligibility, confirmed eligible, included in the study, completing follow-up, and analysed  Figure 1 illustrates the eligibility and inclusion of HIV-infected children and adolescents enrolling in HIV programs. Table 1 describes the profiles of the IeDEA cohort network by geographical regions. |
|  |  | (b) Give reasons for non-participation at each stage  Not applicable |
|  |  | (c) Consider use of a flow diagram  See Figure 1 |
| Descriptive data | 14* | (a) Give characteristics of study participants (eg demographic, clinical, social) and information on exposures and potential confounders  See Results Paragraph 2 and Table 1 |
|  |  | (b) Indicate number of participants with missing data for each variable of interest  Indicated in Figure 3 |
|  |  | (c) Summarise follow-up time (eg, average and total amount)  Presented in Results paragraph 1. |
| Outcome data | 15* | Report numbers of outcome events or summary measures over time  Cumulative incidence of ART initiation for all regions, age group and ART eligibility criteria reported in Figure 2 and Figure S-1. Results paragraphs 3 |
| Main results | 16 | (*a*) Give unadjusted estimates and, if applicable, confounder-adjusted estimates and their precision (eg, 95% confidence interval). Make clear which confounders were adjusted for and why they were included  Results paragraph 5 and 6 Tables 3 and S1. |
|  |  | (*b*) Report category boundaries when continuous variables were categorized  Results Tables 1, 3 S-1 ; Figures 2, S2 and S3 |
|  |  | (*c*) If relevant, consider translating estimates of relative risk into absolute risk for a meaningful time period  Cumulative incidence of outcomes is displayed in Figure 2, S-2 an S-3. |
| Other analyses | 17 | Report other analyses done—eg analyses of subgroups and interactions, and sensitivity analyses  Cumulative incidence estimates for those with missing eligibility information at baseline are presented in supplemental content (Figure S-1). Sensitivity analyses of cumulative incidence estimates and hazard ratio estimates in centers where ART coverage >95% are reported in Tables S1 and Figures S2 and S3 respectively (Results paragraph 7). |
| Discussion | | |
| Key results | 18 | Summarise key results with reference to study objectives  Summarized in Discussion paragraph 1. |
| Limitations | 19 | Discuss limitations of the study, taking into account sources of potential bias or imprecision. Discuss both direction and magnitude of any potential bias  Limitations are presented in Discussion paragraph 4-6. |
| Interpretation | 20 | Give a cautious overall interpretation of results considering objectives, limitations, multiplicity of analyses, results from similar studies, and other relevant evidence  Internal and external comparisons are given in Discussion paragraph 1. Overall interpretation is given in Discussion paragraph 7. |
| Generalisability | 21 | Discuss the generalisability (external validity) of the study results  This is discussed in the limitations section, Discussion paragraph 4. |
| Other information | | |
| Funding | 22 | Give the source of funding and the role of the funders for the present study and, if applicable, for the original study on which the present article is based  See the Funding Statement entered at submission |

*Give information separately for exposed and unexposed groups.

**Note:** An Explanation and Elaboration article discusses each checklist item and gives methodological background and published examples of transparent reporting. The STROBE checklist is best used in conjunction with this article (freely available on the Web sites of PLoS Medicine at http://www.plosmedicine.org/, Annals of Internal Medicine at http://www.annals.org/, and Epidemiology at http://www.epidem.com/). Information on the STROBE Initiative is available at http://www.strobe-statement.org.
