# Supplementary figures and images for: Access to antiretroviral therapy in HIV-infected children aged 0–19 years in the International Epidemiology Databases to Evaluate AIDS (IeDEA) Global Cohort Consortium, 2004–2015: A prospective cohort study
Source: PLoS Med. 2018 May 4;15(5):e1002565. doi: 10.1371/journal.pmed.1002565 (PMC5935422; doi:10.1371/journal.pmed.1002565)

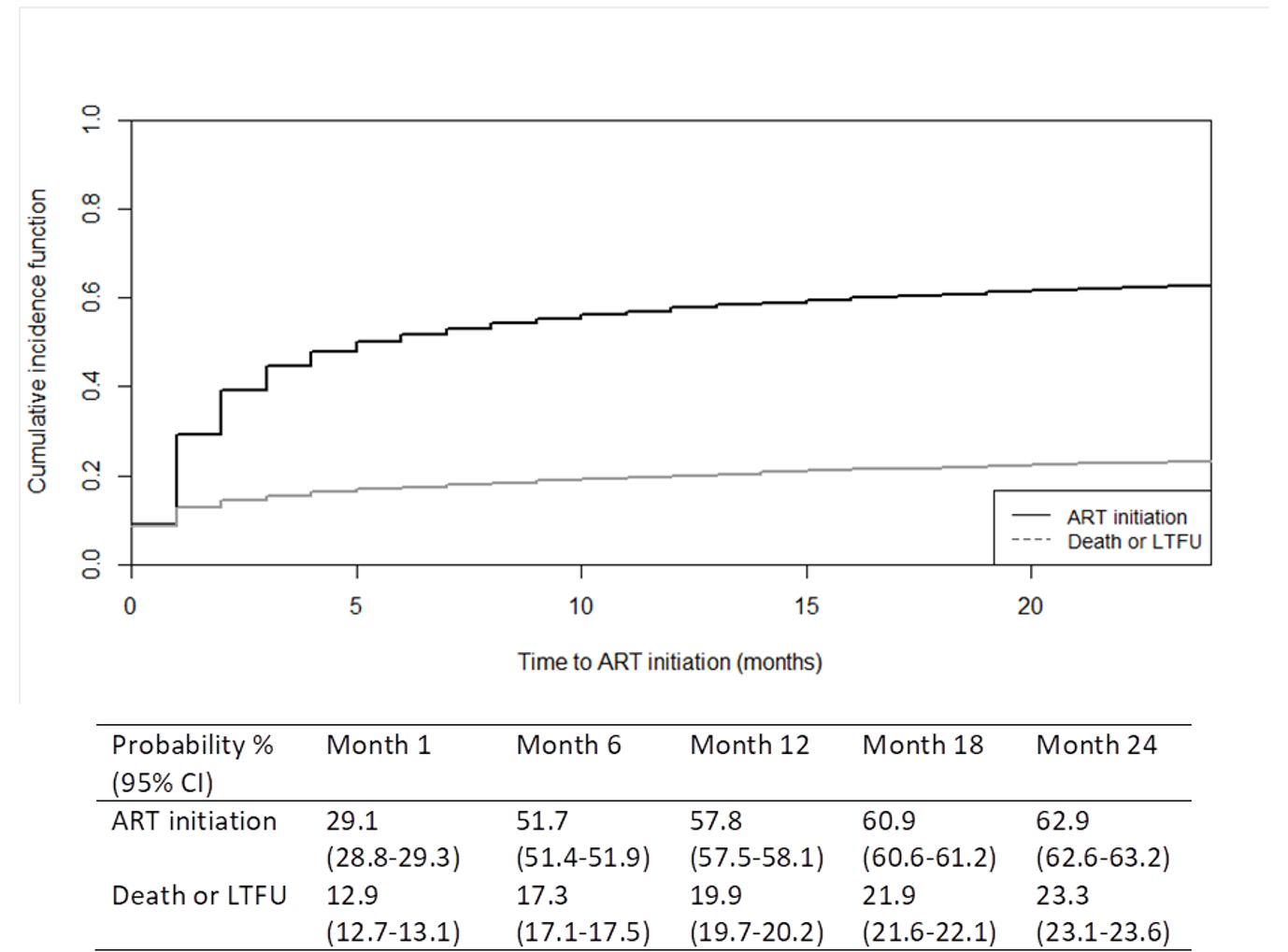

Supplement: S1 Fig — Pediatric IeDEA Global Cohort Consortium, 2004–2015. (TIF) [file pmed.1002565.s002.tif]

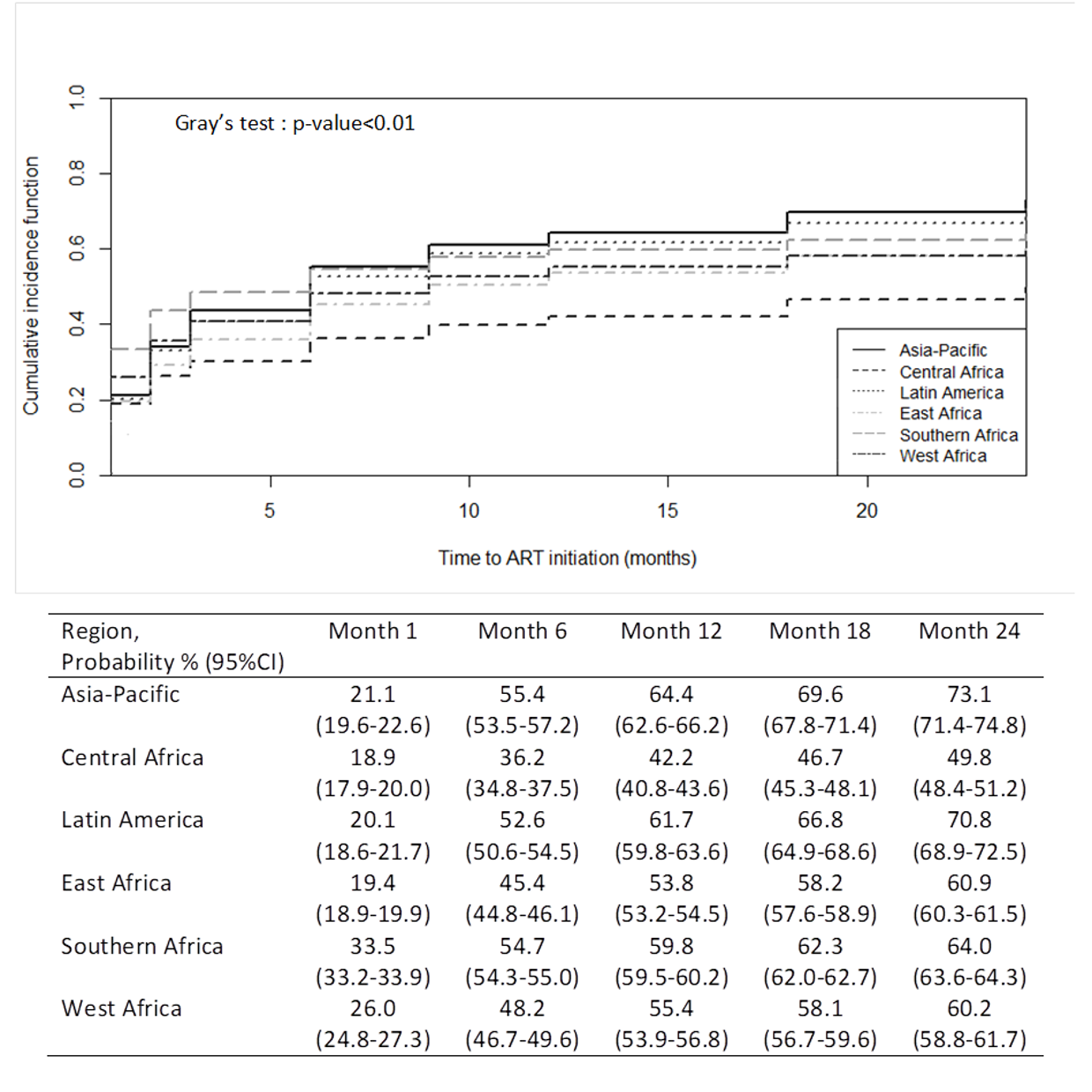

Supplement: S2 Fig — IeDEA Global Cohort Consortium, 2004–2015. (TIF) [file pmed.1002565.s003.tif]

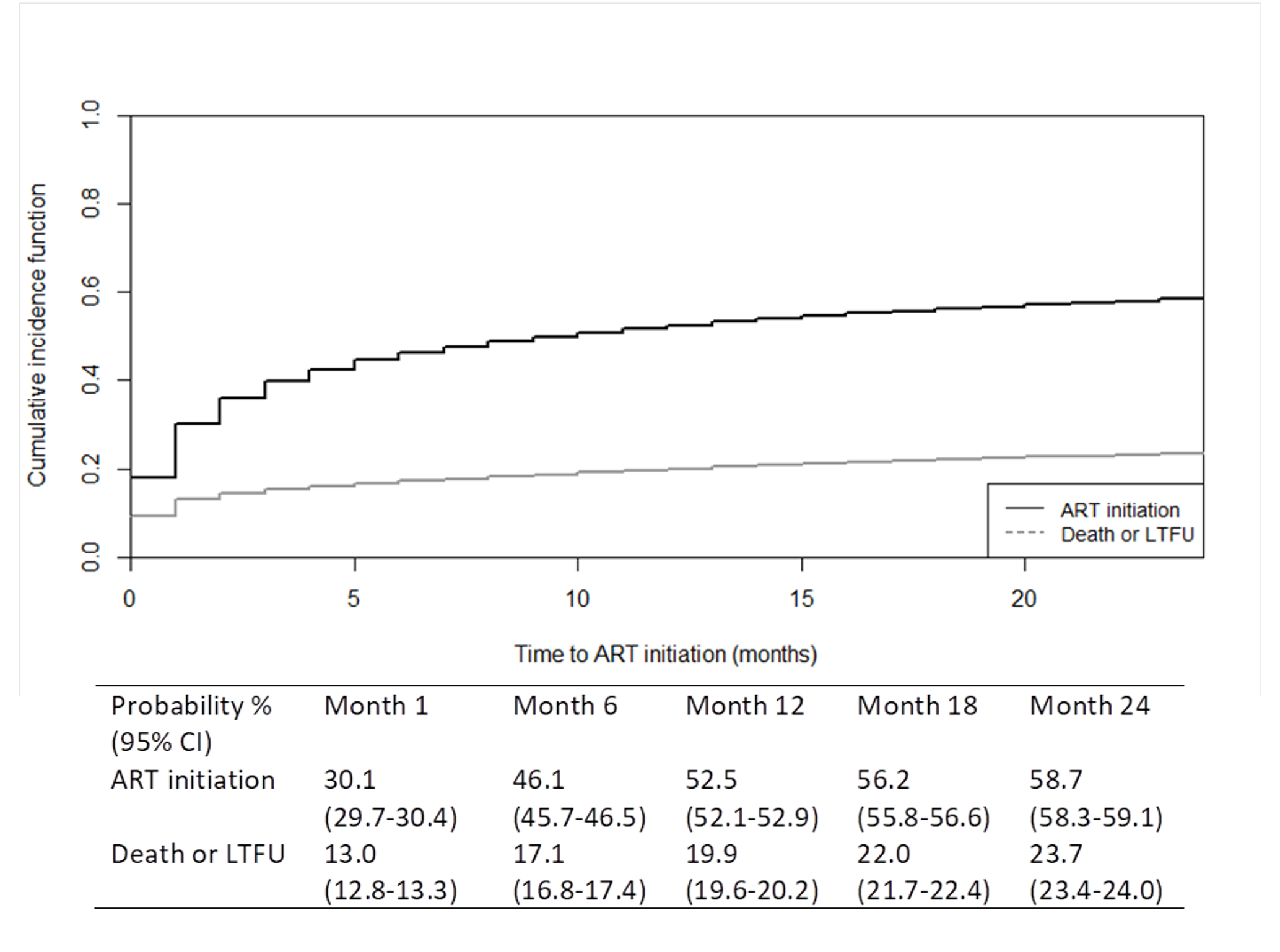

Supplement: S3 Fig — IeDEA Global Cohort Consortium, 2004–2015. (TIF) [file pmed.1002565.s004.tif]
