# Supplementary material for: Access to antiretroviral therapy in HIV-infected children aged 0–19 years in the International Epidemiology Databases to Evaluate AIDS (IeDEA) Global Cohort Consortium, 2004–2015: A prospective cohort study
Source: PLoS Med. 2018 May 4;15(5):e1002565. doi: 10.1371/journal.pmed.1002565 (PMC5935422; doi:10.1371/journal.pmed.1002565)
Supplement: S1 Table — IeDEA Global Cohort Consortium, 2004–2015. (DOCX) [file pmed.1002565.s005.docx]

S-Table 1. Factors associated with ART initiation during pre-ART follow-up in HIV-infected children (n=112,134). The IeDEA Global Cohort Consortium, 2004-2015.

|  | Univariate analysis | | | Full model | | |
| --- | --- | --- | --- | --- | --- | --- |
|  | sHR* | CI (95%)** | *P* | asHR^$^ | CI (95%)** | *P* |
| Gender |  |  | <0.01 |  |  | <0.01 |
| Males | 1 | - |  | 1 | - |  |
| Females | 0.89 | (0.87-0.90) |  | 0.92 | (0.91-0.93) |  |
| Missing | 0.85 | (0.76-0.96) |  | 0.89 | (0.78-1.00) |  |
| Region |  |  | <0.01 |  |  | <0.01 |
| Latin America | 1 | - |  | 1 | - |  |
| Asia-Pacific | 1.00 | (0.95-1.04) |  | 0.85 | (0.81-0.89) |  |
| Central Africa | 0.61 | (0.59-0.64) |  | 0.60 | (0.57-0.63) |  |
| East Africa | 0.73 | (0.71-0.75) |  | 0.70 | (0.67-0.73) |  |
| Southern Africa | 0.82 | (0.79-0.84) |  | 0.80 | (0.77-0.83) |  |
| West Africa | 0.74 | (0.71-0.78) |  | 0.60 | (0.57-0.63) |  |
| Age at baseline |  |  | <0.01 |  |  | <0.01 |
| 0-11 months | 0.70 | (0.68-0.71) |  | 0.77 | (0.75-0.79) |  |
| 12-23 months | 0.83 | (0.81-0.86) |  | 0.84 | (0.82-0.86) |  |
| 2-4 years | 0.84 | (0.82-0.86) |  | 0.86 | (0.84-0.88) |  |
| 5-9 years | 0.88 | (0.86-0.90) |  | 0.97 | (0.95-0.99) |  |
| 10-14 years | 1 | - |  | 1 | - |  |
| 15-19 years | 0.70 | (0.68-0.72) |  | 0.70 | (0.68-0.72) |  |
| Period of enrollment |  |  | <0.01 |  |  | <0.01 |
| < April 2008 | 0.63 | (0.61-0.65) |  | 0.57 | (0.56-0.59) |  |
| [April 2008-July 2010[ | 0.66 | (0.64-0.67) |  | 0.61 | (0.59-0.63) |  |
| [July 2010-June 2013[ | 0.75 | (0.73-0.77) |  | 0.73 | (0.71-0.75) |  |
| ≥ June 2013 | 1 | - |  | 1 | - |  |
| Country income |  |  | <0.01 |  | (-) | <0.01 |
| Upper middle/High | 1 | - |  | 1 | - |  |
| Low/lower middle | 1.12 | (1.10-1.14) |  | 1.22 | (1.20-1.25) |  |
| Clinical^£^ or immunological^***^ eligibility |  |  | <0.01 |  |  | <0.01 |
| At baseline | 1 | - |  | 1 | - |  |
| During follow-up | 0.62 | (0.60-0.63) |  | 0.60 | (0.58-0.61) |  |
| Never | 0.43 | (0.42-0.45) |  | 0.45 | (0.44-0.48) |  |
| Missing | 0.59 | (0.58-0.60) |  | 0.56 | (0.55-0.57) |  |

*sHR = sub-distribution hazard ratio, **CI = confidence interval, ^$^asHR = adjusted sub-distribution hazard ratio; ^***^ Severe immunodeficiency for age: CD4 ≤25% if age <5 years or CD4≤350 cells/µl if age≥5 years; ^£^ Clinical stage III, IV or AIDS;
