# Supplementary material for: Access to antiretroviral therapy in HIV-infected children aged 0–19 years in the International Epidemiology Databases to Evaluate AIDS (IeDEA) Global Cohort Consortium, 2004–2015: A prospective cohort study
Source: PLoS Med. 2018 May 4;15(5):e1002565. doi: 10.1371/journal.pmed.1002565 (PMC5935422; doi:10.1371/journal.pmed.1002565)
Supplement: S1 Concept Plan — (PDF) [file pmed.1002565.s007.pdf]

International Epidemiologic Databases to Evaluate AIDS (IEDEA)

CONCEPT SHEET FOR MULTI-REGIONAL PROJECTS (to be written in English)

**Title:**	**IedEA-WHO collaboration: global analysis of the pre-ART cascade and delay from diagnosis to start of antiretroviral therapy in HIV-infected children aged 0-19 years.**<br>Concept number #2.037
**Project PI:**	Valériane Leroy (Lead), François Dabis (PI), West Africa
**IeDEA correspondents (working group members):**	Working group for concept sheet design, with authorship of individual papers to be determined (please see deliverables below): TBD
**Collaborators (working group members):**	All IeDEA regions will be invited to collaborate and to contribute data
**Data manager:**	West Africa: Karen Malateste
**Statistician:**	West Africa: Sophie Desmonde, Karen Malateste
**Where will data be merged?**	Inserm U1219, Bordeaux, France
**Where will statistical analyses be done?**	Data will be aggregated and analyzed by the IeDEA West African team (Karen Malateste)
**Abstract:**<br>(about 200 words)	**Background:** In low- and middle-income countries, the attrition across the continuum of care of HIV infected children is not well known, but needed to reach the 90-90-90 target. Particularly in young children, early ART initiation is slowed down by the operational difficulties of early infant diagnosis based on virological testing. These difficulties lead to important pre-ART delays competing with high pre-ART-mortality. This project is a unique opportunity to provide a global analysis of the pre-ART retention cascade in children within the IeDEA network in order to better monitor the ART roll-out worldwide.<br>**Objectives**: to provide a global analysis within the IeDEA network from 2004 to 2015 characterizing:<br>1/ HIV-infected children (0-19 years at inclusion), for each calendar year since 2004 to 2015, overall and stratified by country income group, region, gender, CD4 cell count, CD4% and age (<1 year, 1-2, 3-4 years, 5-9 years, 10-15 years and 15-19 years) and evolving WHO ART eligibility criteria at time of their HIV diagnosis, enrolment into the pediatric HIV programs, and ART initiation;<br>2/ the proportion of children enrolled into programs after their HIV diagnosis and the missed opportunities for inclusion (death, lost-to-follow-up (LTFU), transferred out) for each calendar year since 2004 to 2015.<br>3/ the proportion of children initiated on ART after their enrolment into programs and the missed opportunities for ART initiation (death, lost-to-follow-up, transferred out).<br>4/ the delays between HIV diagnosis, enrollment into programs and ART initiation for each calendar year since

	2004 to 2015.<br>5/ the cumulative incidence of children initiated on ART among children diagnosed, then enrolled into HIV programs, taking death, then death or LTFU as competing risk events at 1, 2, 3,6, 12, 18, 24 months after HIV diagnosis/HIV enrollment into programs.<br>6/ the correlates of ART initiation accounting for death and LTFU as a competing risk until 24 months.<br>7/ the fist-line regimen initiated.<br><br>**Methods:**<br><u>Study sites</u>: All pediatric IeDEA clinical centres with children aged 0-19 years at inclusion.<br><u>Data</u>**:** Region**,** country, sites, demographics (sex; date of birth, date of HIV diagnosis if available and enrolment in care); clinical WHO/CDC staging at enrolment and ART initiation; laboratory values and dates (CD4 cell count, CD4 percent); prophylaxis (date of cotrimoxazole start); date of ART initiation; regimen of ART initiation; date of death; date of LTFU, date of transfer out.<br>**Anticipated results:** Number and percent of children enrolled into HIV care among those diagnosed HIV-infected; number and percent of children initiated on ART among those enrolled; yearly cumulative incidence curves of ART-initiation with death then death/LTFU, as competing risk events will be computed for each calendar year overall, and stratified by country income, region, sex, age groups, CD4 cell count.<br>**Significance**: This concept sheet is part of the IeDEA-WHO collaboration project and will be useful to guide WHO guidelines.
	**Background**<br>The Global Plan towards the elimination of new pediatric infections has made substantial progress since it was first announced in 2011, with a 43% decline of new pediatric HIV infections overall between 2009 and 2013 among the 21 eMTCT priority countries. However, in 2013 there remained an estimated 240,000 infants born with HIV worldwide (1). Without antiretroviral treatment, 35% of children living with HIV die by their first birthday and 52% die by the end of their second year (2). In 2008, the CHER trial demonstrated that early antiretroviral therapy (ART) before the 12th week of life reduces HIV-related mortality in children living with HIV by 75% (3). Consequently, the World Health Organization (WHO) revised treatment recommendations so that systematic ART would be initiated for all HIV-infected children under the age of 12 months and systematic early infant diagnosis carried out for all exposed children from 6 weeks of age. These recommendations were revised extending immediate ART initiation to all children less than 24 months regardless of any clinical or immunological criteria (in 2010) and then to all those 5 years in 2013; treatment initiation in older children is recommended according to clinical or immunological criteria (WHO clinical stage 3 or 4 disease or CD4 ≤ 500 cells/µL) (4).<br><br>Clinical care and access to ART for HIV-infected children in resource-limited settings is a complex problem to analyze, with

	many operational issues. Although access to ART is improving, it remains insufficient with only 22% of eligible children receiving treatment in the 21 priority countries (1). A major challenge of health care programs in the context of rapid scale-up of ART is to initiate children on ART after they have tested positive for HIV. While early HIV infant diagnosis (EID) could be delayed substantially in children< 18 months in resources-limited settings (5, 6), the period between HIV diagnosis and ART initiation can also extend for several additional months, as CD4 count has to be determined (for those >5 years only since 2013) and caregivers have to go through the process of pre-treatment counselling (7). For children, this period between HIV diagnosis and initiation on ART is critical because of a more rapid disease progression to early death than in adults (8, 9). Studies of retention in pre-ART care report substantial loss of patients at every step, starting with patients who do not return for their initial CD4 count results and ending with those who do not initiate ART despite eligibility in adults (10). However, few data exist on loss-to-program in the pre-ART cascade in children. In 2011, a systematic review reported on pre-ART retention in care: the percentage of children diagnosed with HIV who benefited from a CD4 cell measurement ranged from 78% to 97% at the median age of 2.2 to 6.5 years. Among these children, the proportion of those eligible for ART ranged from 63% to 89%, and the proportion of children actually initiating treatment ranged from 39% to 99% (11). Barriers to ART initiation have been described in various settings and reasons for missing appointments include stigmatization, lack of human resources with long queues and overloaded clinics (12-17). As a result, although findings differed substantially across IeDEA regions, the same concerns were raised everywhere about delayed ART start, and low access to free HIV services for children, with increased workload over time in lower-income countries (18). These difficulties lead to important pre-ART delays competing with high pre-ART-mortality.<br><br>Monitoring linkages across the cascade of pediatric HIV care from HIV-positive diagnosis through entry into care, and initiation of ART, retention and viral suppression is essential to ensure that pediatric ART programs are generally improving children's health. Pre-ART refers to the periods between HIV diagnosis, enrolment in HIV care programs and ART initiation. This project is a unique opportunity to provide a global analysis of the pre-ART retention cascade in children within the IeDEA network in order to better monitor the ART roll-out worldwide.<br><br>**Objectives**: to provide a global analysis from 2004 to 2015 within the IeDEA network characterizing:<br>1/ HIV-infected children (0-19 years at inclusion), overall and for each calendar year, country income group, region, gender, WHO/CDC clinical staging, CD4 cell count, CD4%, and age (<1 year, 1-2, 3-4 years, 5-9 years, 10-15 years and 15-19 years) and evolving WHO ART eligibility criteria at time of their HIV diagnosis if available, enrolment into the pediatric HIV programs, and ART initiation;<br>2/ the proportion of children enrolled into HIV care programs

	after their HIV diagnosis and the missed opportunities for inclusion (death, lost-to-follow-up [LTFU], transferred out).<br>3/ the proportion of children initiated on ART after their enrolment into programs and the missed opportunities for ART initiation (death, LTFU, transferred out).<br>4/ the delays between HIV diagnosis, enrollment into programs and ART initiation.<br>5/ the cumulative incidence of children initiated on ART among children diagnosed, then enrolled into HIV programs, taking death, then death or LTFU as competing risk events at 1, 2, 3,6, 9,12, 18, 24 months after HIV diagnosis/HIV enrollment into programs.<br>6/ the correlates of ART initiation accounting for death and LTFU as a competing risk until 24 months.<br>7/ the fist-line regimen initiated.<br><br>We hope that these data will both help inform global ART reports surveillance and WHO guidelines.<br>**Study design**: Multiregional IeDEA cohort part of the IeDEA-WHO collaboration project.<br>**Study sites**: All clinical centres from IeDEA Collaboration regions taking care of children aged 0-19 years.<br><br>**Eligibility criteria**<br>All HIV-1-infected-chidren, aged < 19 years at enrolment into HIV care programs regardless of whether they were initiated on ART.<br>**Exclusion criteria**<br>Children not ART-naïve at inclusion.<br><br>**Data**<br>Data that will be needed are outlined in the data transfer request enclosed.<br>Region**,** country, sites, demographics (sex; date of birth, date of HIV diagnosis if available and enrolment in care); clinical WHO/CDC staging at enrolment and ART initiation; laboratory values and dates (CD4 cell count, CD4 percent); prophylaxis (date of cotrimoxazole start); date of ART initiation; date of death; date of LTFU, date of transfer out, first ARV regimen.<br><br>**Outcomes**: for each calendar year from 2004 to 2015, overall then, stratified by country income, region, sex, age groups, CD4 cell count.<br>• Number and proportion of children enrolled into HIV care among those diagnosed as HIV-infected;<br>• Number and proportion of children initiated on ART among those enrolled (defined by at least a three-drugs regimen);<br>• Yearly cumulative incidence curves of ART-initiation among children diagnosed/enrolled into HIV care with death and LTFU as competing risk will be computed;<br>• Time to HIV care enrolment since HIV diagnosis when available;<br>• Time to ART initiation since enrolment into HIV programs /HIV diagnosis;

	• Pre-ART mortality rates among children diagnosed/enrolled into HIV care;<br>• Pre-ART LTFU rates among children diagnosed/enrolled into HIV care;<br>**Statistical methods**<br>Descriptive statistics will be used to estimate proportion of death, LTFU, and describe time between HIV diagnosis, enrolment into HIV care, eligibility for ART according to WHO guidelines at time of inclusion and ART initiation, for the subset with available diagnosis information.<br><br>Cumulative incidence curves will be used to describe the proportion of children on ART within pre-defined time periods since HIV/diagnosis/enrolment into HIV care (1, 2, 3, 6, 9,12,18, 24 months after HIV diagnosis/HIV enrollment into programs) and since treatment eligibility for those children > 5 years of age (1, 3, 6 and 12 months).<br><br>Determinants of ART initiation, pre-ART mortality and LTFU since HIV diagnosis/enrolment will be described in survival analyses, using a competing risk approach. The principal outcome will be ART initiation; pre-ART mortality and LTFU are competing events.<br><br>Loss to follow-up (defined as last follow-up to date of database closure delay greater than 6 months):<br>CD4 at inclusion, and at ART start : -3 months/+1 week around the date.<br><br>**Sample size considerations**<br>Number of children will depend on sites with access to pre-enrolment and pre-ART data. No sample was computed for this analysis.<br><br>**Ethical considerations**<br>Each participating pediatric HIV clinic formally agreed to be included in the IeDEA collaboration, with a local Institutional Review Board and NIH approval to contribute data to IEDEA collaborative analysis.<br><br>**Deliverables**<br>Report to the IeDEA-WHO collaboration group.<br>We anticipate one clinical paper from this concept sheet: Time to ART initiation since HIV diagnosis in HIV-infected children in the IeDEA network.
**Funding:**	None
**Target journals:**	Supporting WHO 2016 global HIV progress report
**Milestones:**	By early-may: Regions leading an analysis circulate their multi-regional concept sheets by email to the IeDEA Executive Committee for feedback and approval.<br>By mid-May: Multi-regional data request forwarded to the

	regional data manager.<br>By mid-July: Analyses for the IeDEA-WHO collaboration completed.<br>By end of June : analyses conducted<br>By end-July: Analyses reports submitted to WHO.

References

1. World Health O. Global AIDS response progress reporting 2014. 2014 February 2014. Report No.
2. Newell M-L, Coovadia H, Cortina-Borja M, Rollins N, Gaillard P, Dabis F. Mortality of infected and uninfected infants born to HIV-infected mothers in Africa: a pooled analysis. Lancet. 2004;364(9441):1236-43.
3. Violari A, Cotton MF, Gibb DM, Babiker AG, Steyn J, Madhi SA, et al. Early antiretroviral therapy and mortality among HIV-infected infants. N Engl J Med. 2008;359(21):2233-44.
4. World Health Organisation. Consolidated guidelines on the Use of Antiretroviral Drugs for Treating and Preventing HIV Infection. Recommendations for a public health approach. Geneva: WHO, UNAIDS; 2013 [updated 30 june 2013]. Available from: http://www.who.int/hiv/pub/guidelines/arv2013/download/en/index.html.
5. Ciaranello AL, Park JE, Ramirez-Avila L, Freedberg KA, Walensky RP, Leroy V. Early infant HIV-1 diagnosis programs in resource-limited settings: opportunities for improved outcomes and more cost-effective interventions. BMC Med. 2011;9:59.
6. Penazzato M, Revill P, Prendergast AJ, Collins IJ, Walker S, Elyanu PJ, et al. Early infant diagnosis of HIV infection in low-income and middle-income countries: does one size fit all? Lancet Infect Dis. 2014.
7. Zachariah R, Tayler-Smith K, Manzi M, Massaquoi M, Mwagomba B, van Griensven J, et al. Retention and attrition during the preparation phase and after start of antiretroviral treatment in Thyolo, Malawi, and Kibera, Kenya: implications for programmes? Trans R Soc Trop Med Hyg. 2011;105(8):421-30.
8. Newell ML, Coovadia H, Cortina Borja M, Rollins N, Gaillard P, Dabis F, et al. Mortality of infected and uninfected infants born to HIV-infected mothers in Africa: a pooled analysis. Lancet. 2004;364(9441):1236-43.
9. Okomo U, Togun T, Oko F, Peterson K, Jaye A. Mortality and loss to programme before antiretroviral therapy among HIV-infected children eligible for treatment in The Gambia, West Africa. AIDS Res Ther. 2012;9(1):28.
10. Rosen S, Fox MP. Retention in HIV Care between Testing and Treatment in Sub-Saharan Africa: A Systematic Review. PLos Med. 2011;8(7):e1001056-e.
11. Mugglin C, Wandeler G, Estill J, Egger M, Bender N, Davies MA, et al. Retention in care of HIV-infected children from HIV test to start of antiretroviral therapy: systematic review. PLoS ONE. 2013;8(2):e56446.
12. Kurewa NE, Gumbo FZ, Mapingure PM, Munjoma MW, Chirenje MZ, Rusakaniko S, et al. Predictors of attrition among children born in a PMTCT programme in Zimbabwe followed up over 5 years. J Trop Pediatr. 2012;58(5):360-9.
13. Yeap AD, Hamilton R, Charalambous S, Dwadwa T, Churchyard GJ, Geissler PW, et al. Factors influencing uptake of HIV care and treatment among children in South Africa - a qualitative study of caregivers and clinic staff. AIDS Care. 2010;22(9):1101-7.
14. Phelps BR, Ahmed S, Amzel A, Diallo MO, Jacobs T, Kellerman SE, et al. Linkage, initiation and retention of children in the antiretroviral therapy cascade: an overview. AIDS. 2013;27 Suppl 2:S207-13.
15. Vreeman RC, Nyandiko WM, Ayaya SO, Walumbe EG, Marrero DG, Inui TS. The perceived impact of disclosure of pediatric HIV status on pediatric antiretroviral therapy adherence, child well-being, and social relationships in a resource-limited setting. AIDS Patient Care STDS. 2010;24(10):639-49.
16. Brouwer CN, Lok CL, Wolffers I, Sebagalls S. Psychosocial and economic aspects of HIV/AIDS and counselling of caretakers of HIV-infected children in Uganda. AIDS Care. 2000;12(5):535-40.
17. Coulibaly M, Meda N, Yonaba C, Ouedraogo S, Congo M, Barry M, et al. Missed Opportunities for Early Access to Care of HIV-Infected Infants in Burkina Faso. PLoS ONE. 2014;9(10):e111240.
18. Leroy V, Malateste K, Rabie H, Lumbiganon P, Ayaya S, Dicko F, et al. Outcomes of antiretroviral therapy in children in Asia and Africa: a comparative analysis of the IeDEA pediatric multiregional collaboration. J Acquir Immune Defic Syndr. 2013;62(2):208-19.
